# Ultralarge Free‐Standing Imine‐Based Covalent Organic Framework Membranes Fabricated via Compression

**DOI:** 10.1002/advs.202104643

**Published:** 2022-01-17

**Authors:** Jesús Á. Martín‐Illán, José Antonio Suárez, Julio Gómez‐Herrero, Pablo Ares, Daniel Gallego‐Fuente, Youdong Cheng, Dan Zhao, Daniel Maspoch, Félix Zamora

**Affiliations:** ^1^ Departamento de Química Inorgánica Universidad Autónoma de Madrid Madrid 28049 Spain; ^2^ Catalan Institute of Nanoscience and Nanotechnology (ICN2) CSIC and BIST Campus UAB Bellaterra Barcelona 08193 Spain; ^3^ Departamento de Física de la Materia Condensada Universidad Autónoma de Madrid Madrid 28049 Spain; ^4^ Condensed Matter Physics Center (IFIMAC) Universidad Autónoma de Madrid Madrid 28049 Spain; ^5^ Department of Chemical and Biomolecular Engineering National University of Singapore 4 Engineering Drive 4 Singapore 117585 Singapore; ^6^ ICREA Pg. Lluís Companys 23 Barcelona 08010 Spain; ^7^ Instituto Madrileño de Estudios Avanzados en Nanociencia (IMDEA‐Nanociencia) Cantoblanco Madrid 28049 Spain; ^8^ Institute for Advanced Research in Chemical Sciences (IAdChem) Universidad Autónoma de Madrid Madrid 28049 Spain

**Keywords:** aerogels, COF‐membranes, covalent organic frameworks, gas mixtures separation, porous materials

## Abstract

Demand continues for processing methods to shape covalent organic frameworks (COFs) into macroscopic objects that are needed for their practical applications. Herein, a simple compression method to prepare large‐scale, free‐standing homogeneous and porous imine‐based COF‐membranes with dimensions in the centimeter range and excellent mechanical properties is reported. This method entails the compression of imine‐based COF‐aerogels, which undergo a morphological change from an elastic to plastic material. The COF‐membranes fabricated upon compression show good performances for the separation of gas mixtures of industrial interest, N_2_/CO_2_ and CH_4_/CO_2_. It is believed that the new procedure paves the way to a broader range of COF‐membranes.

## Graphical abstract

Large‐scale free‐standing covalent organic framework (COF)‐membranes are prepared via a simple compression method using imine‐based COF‐aerogel compression. This straightforward method produces free‐standing and homogeneous COF‐membranes with sizes in the centimeter range and excellent mechanical properties. They show good performance for gas separation of mixtures of industrial interest.

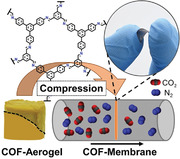

## 1 Introduction

The fabrication of efficient membranes is crucial for the separation processes found in myriad applications. In this context, membrane technology is evolving fast to offer straightforward and more ecofriendly solutions with lower energy consumption than conventional separation processes.^[^ ^1^ ^]^ In the last years, the development of new materials has played a key role in advancing membrane separation technology. Among the different materials, graphene, graphene oxide, and other alternative 2D materials have certainly played a significant role in membrane development.^[^ ^2^ ^]^ While these materials do not show intrinsic porosity, the tortuous transport pathways formed between these nanosheets allow the size‐specific permeation of molecules.^[^ ^3^ ^]^ Alternatively, other porous materials such as classical inorganic‐based materials (e.g., zeolites, silicas, or carbon)^[^ ^4^ ^]^ and more novel organic‐based materials (e.g., conjugated microporous polymers (CMPs),^[^ ^5^ ^]^ metal–organic frameworks (MOFs),^[^ ^6^ ^]^ and polymers of intrinsic microporosity (PIMs)^[^ ^7^ ^]^) have been incorporated in the fabrication of membranes because of their advantages (i.e., simultaneous high permeability and high selectivity) over traditional organic polymeric membranes.^[^ ^8^ ^]^

Covalent organic frameworks (COFs) comprise an emerging class of porous materials that integrates organic subunits into periodic 2D or 3D crystalline structures held together by strong covalent bonds.^[^ ^9^ ^]^ COFs are thermally and chemically stable in harsh environments, such as extreme humidity, strong acids, and organic solvents.^[^ ^10^ ^]^ Moreover, in contrast to classical 2D materials in which their permeable pathways rely only on interlamellar transport and/or deliberate introduction of in‐plane defects, 2D‐COFs also display ordered in‐plane pores. In addition, COFs allow accurate and predictable control over composition, topology, and porosity. In this context, imine‐based COFs are an excellent choice for many applications^[^ ^11^ ^]^ since they can also be processed into different macroscopic morphologies, including foams, aerogels, and membranes.^[^ ^12^ ^]^

Before this work, several methods have been developed to fabricate COFs and COF‐based membranes, which have already demonstrated remarkable applications in catalysis and separation processes.^[^ ^11c^ ^]^ Some of the most common fabrication methods include COF blending into polymer matrices to form mixed‐matrix membranes (MMMs)^[^ ^13^ ^]^ and the in situ growth method.^[^ ^14^ ^]^ Recently, new ways have been reported to produce continuous COF‐membranes, either on surfaces or free‐standing.^[^ ^15^ ^]^ The first approach used the conventional solvothermal synthesis of COFs on the surface of solid substrates.^[^ ^16^ ^]^ For the fabrication of free‐standing COF‐membranes, a widely explored method is interfacial polymerization. This fabrication method occurs at the interface of liquid/liquid^[^ ^17^ ^]^ or liquid/gas.^[^ ^18^ ^]^ However, it is limited to laboratory scale because it is a tedious procedure that requires slow monomer diffusion between two phases, leading to crystalline polymerization. Additionally, the so‐formed membrane must be transferred before its use, making its real application hampered for large‐scale preparation. Alternatively, an approach based on solution casting and baking was reported to transform the molecular precursors into COF‐membranes. This process allows the fabrication of COF‐membranes with dimensions in the hundreds of microns. However, it is so far limited to beta‐ketoenamine COFs.^[^ ^19^ ^]^ More recently, Jiang et al. developed a solution‐processing method,^[^ ^20^ ^]^ in which large‐area COF‐membranes are fabricated by exposing an amorphous polymeric membrane to a monomer exchange process under solvothermal conditions.

Herein, we show that large‐scale free‐standing COF‐membranes can be prepared via a simple compression method. Our method to produce COF‐membranes started from observation during a mechanical study of our recently reported imine‐based COF‐aerogels.^[^ ^21^ ^]^ These COF‐aerogels showed to behave elastically below 25–35% strain. However, further compression to a maximum deformation of 90% gave rise to a plastic behavior without exhibiting failure, with partial recovery, even at maximum deformation. We, therefore, postulated that control of this compression process in COF‐aerogels could become a straightforward method to produce free‐standing, homogeneous COF‐membranes with sizes in the centimeter range and excellent mechanical properties. We anticipate that these membranes show good performance for gas separation of mixtures of industrial interest, e.g., N_2_/CO_2_ and CH_4_/CO_2_.

## 2 Results and Discussion

The fabrication of COF‐membranes started with the preparation of three COF‐aerogels, TAPB‐BTCA‐AGCOF, PPDA‐BTCA‐AGCOF, and TAPB‐PDA‐AGCOF, following our previously reported procedure.^[^ ^21^ ^]^ Then, these COF‐aerogels were gently broken into smaller pieces (size: ≈1–3 mm) in the presence of AcOH (5 µL of AcOH for 10 mg of COF). Finally, a fixed amount of the resulting pieces (0.0037 g cm^−2^) was pressed under 120 MPa for 5 min to produce free‐standing membranes of the corresponding COFs; hereafter named as TAPB‐BTCA‐MCOF, PPDA‐BTCA‐MCOF, and TAPB‐PDA‐MCOF.

**Figure 1:**
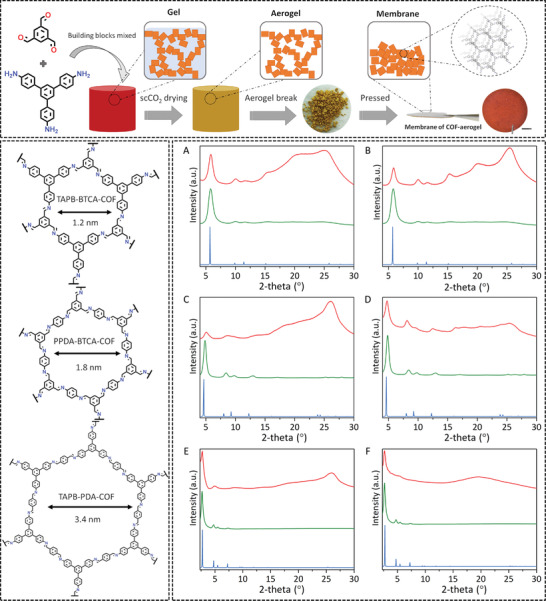
Top: Schematic representation of the synthesis (sol–gel processes) of a COF‐aerogel and its processing to a free‐standing membrane. This method comprises i) mixture of the molecular building blocks in AcOH; ii) formation of the COF gels; iii) solvent exchange and supercritical CO_2_ activation; iv) breaking process; and v) pressing of the COF‐aerogel pieces. Photograph corresponds to a TAPB‐BTCA‐MCOF‐membrane. Scale bar: 0.3 cm. Bottom left: Representation of the structures of the 2D imine‐based COFs used in this work. Bottom right: A,C,E) Reflection and B,D,F) transmission PXRD patterns of the corresponding membranes (red), aerogels (green), and simulated patterns (blue) for A,B) TAPB‐BTCA‐COF, C,D) PPDA‐BTCA‐COF, and E,F) TAPB‐PDA‐AGCOF.

The crystalline structure of COF‐membranes was confirmed by powder X‐ray diffraction (PXRD) in reflection (parallel) and transmission (perpendicular) mode and deduced by theoretical simulation.^[^ ^22^ ^]^ Indeed, the different membranes exhibited good crystallinity, comparable with the PXRD patterns of their COF‐aerogels counterparts and matching the simulated patterns of the AA‐eclipsed stacking models. Thus, in parallel mode, TAPB‐BTCA‐MCOF showed an intense peak at 5.7° corresponding to the (100) plane, along with the peaks at 9.9°, 11.5°, and 25.3° attributed to the (110), (200), and (001) reflections (**Figure** **1**A). As the pore size increases, the peak corresponding to the (100) plane gets shifted to lower 2*θ* values of 4.8° for PPDA‐BTCA‐MCOF (Figure 1C) and 2.9° for TAPB‐PDA‐MCOF (Figure 1E). Thus, the corresponding (001) reflection peak displayed a high‐intensity increase. In contrast, the (100) reflection peak showed a significant attenuation. On the other hand, in perpendicular mode (turning the pellet 90°), the corresponding (*hk*0) peaks displayed a slight increase of intensity (Figures 1B,D,F). We attributed this behavior to a certain degree of preferred orientation of the COFs as showed by a higher intensity of the (100) reflection peak in the perpendicular mode due to the pressure applied during the formation of the COF‐membranes. Nevertheless, this preferred crystallographic orientation is considerably lower than those observed for other imine‐based COFs showing stacking.^[^ ^23^ ^]^ Here, it is also important to mention that we tested the influence of pressure on thickness and crystallinity. We observed that higher pressures (>360 MPa) induce a decrease of crystallinity in the COF‐membranes (Figure S1, Supporting Information). Moreover, we found that the initially reported density values of the COF‐aerogels (≈0.02 g cm^−3^) changed to values ≈1 g cm^−3^, which are in the range of those expected for monocrystalline COF structures.

**Figure 2:**
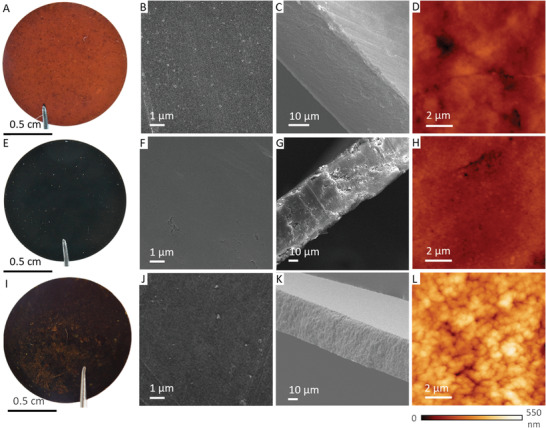
A,E,I) Photographs, B,C,F,G,J,K) SEM micrographs, and D,H,L) atomic force microscope topographies of A–D) TAPB‐BTCA‐MCOF, E–H) PPDA‐BTCA‐MCOF, and I–L) TAPB‐PDA‐MCOF.

Having demonstrated the possibility to fabricate COF‐membranes using this simple compression method, we then scaled it up to produce membranes with diameters ranging from 1 to 5 cm (**Figure** **2** and Figure S2, Supporting Information). Field‐emission scanning electron microscopy (SEM) images showed the formation of COF‐membranes with thicknesses in the range of 50–60 µm (Figure 2), which are thinner than those previously obtained from their COF‐powder counterparts.^[^ ^24^ ^]^ Here, we hypothesize that the lower density of COF‐aerogels compared to their COF‐powder counterparts is crucial for forming thinner COF‐membranes. Moreover, the use of COF‐aerogels revealed the formation of continuous, homogeneous, and compact membranes (Figures 2B,F,J). The surface roughness of these membranes was measured by atomic force microscope (AFM) (Figures 2D,H,L), using WSxM software for acquisition and analysis.^[^ ^25^ ^]^ We determined an average surface roughness of 31 ± 8, 25 ± 4, and 70 ± 7 nm for TAPB‐BTCA‐MCOF, PPDA‐BTCA‐MCOF, and TAPB‐PDA‐MCOF, respectively. These images agreed with the expected material densification that occurs upon pressure, allowing the production of free‐standing COF‐membranes.

We also characterized the COF‐membranes by Fourier transform infrared (FT‐IR) spectroscopy, ^13^C CP‐MAS solid‐state NMR, and thermogravimetric analysis (TGA) under N_2_ atmosphere. The spectroscopic features of the COF‐membranes were almost identical to those of their corresponding COF‐aerogels. The main difference was ascribed to the presence of AcOH guest molecules trapped in the porous structure of the membranes. The appearance of the typical stretching band at ≈1620 cm^−1^ confirmed no change of the imine bond (C═N) (Figures S3–S5, Supporting Information). Furthermore, the characteristic vibrations of the amino and carbonyl groups of the initial monomers were detected, suggesting the presence of unreacted groups likely placed at the defective edges of nanolayers. As we have previously reported,^[^ ^24^ ^]^ the unreacted amine and/or aldehyde groups can connect COF‐polymine nanolayers mediated by AcOH molecules by H‐bonding formation with the amine and aldehyde groups. The AcOH stretching bands were also observed at 1698 cm^−1^ (Figures S3–S5, Supporting Information). Additionally, ^13^C CP‐MAS solid‐state NMR spectra confirmed the formation of imine bonds (≈156 ppm) (Figures S6–S8, Supporting Information). Finally, TGA under N_2_ atmosphere showed that the COF‐membranes are thermally stable up to 500 °C (Figures S9–S11, Supporting Information). From this analysis, one guest molecule of AcOH per formula unit was estimated, as also corroborated by elemental analysis (Table S1, Supporting Information).

Next, the chemical stability of the COF‐membranes was evaluated upon their immersion in acidic, neutral, and basic (pH = 1–14) aqueous solutions and common organic solvents. Analysis of the treated membranes showed no significant changes by PXRD, confirming their high chemical stability (Figure S12, Supporting Information).

We also studied their mechanical properties by performing indentation experiments using atomic force microscopy (AFM). We acquired a force versus distance curve in each pixel of the image and determined Young's moduli of the films by fitting the corresponding indentation curves to the Hertz model.^[^ ^26^ ^]^ We obtained Young's modulus of 0.8 ± 0.3, 0.5 ± 0.2, and 0.4 ± 0.2 GPa for TAPB‐BTCA‐MCOF, PPDA‐BTCA‐MCOF, and TAPB‐PDA‐MCOF, respectively (Figures S13 and S14, Supporting Information). A similar tendency was already observed in the mechanical properties of imine‐based COF‐aerogels, in which the enhancement of the pore size correlated to the fragility of the framework due to the decrease of *π*–*π* stacking force.^[^ ^27^ ^]^

N_2_ gas adsorption experiments at 77 K were performed to evaluate the permanent porosity of all COF‐membranes. The isotherms of the membranes were very similar to those obtained from their aerogel counterparts (Figures S17–S19, Supporting Information).^[^ ^21^ ^]^ From the aforementioned isotherms, we calculated Brunauer−Emmett−Teller surface areas (SA_BET_) of 247 m^2^ g^−1^ for TAPB‐BTCA‐MCOF, 186 m^2^ g^−1^ for PPDA‐BTCA‐MCOF, and 170 m^2^ g^−1^ for TAPB‐PDA‐MCOF. Despite being still porous, these values confirmed a loss of porosity when COF‐aerogels were compressed to produce the corresponding COF‐membranes. This behavior is somehow expected after the membrane formation, where a “gate‐closing” effect can occur, as reported by Zhong et al.^[^ ^28^ ^]^ However, the pore size distribution obtained using the DFT method (Figures S20–S22, Supporting Information) revealed that COF‐membranes had crystallographic pores of the same size as their COF‐aerogels counterparts.

**Figure 3:**
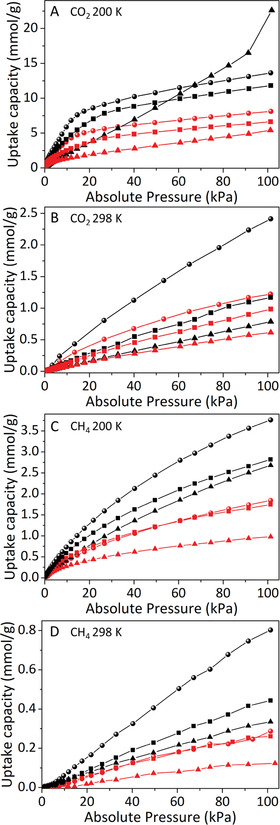
A,B) CO_2_ and C,D) CH_4_ sorption isotherms of aerogels (black) and membranes (red) of TAPB‐BTCA‐COF (sphere‐symbol), PPDA‐BTCA‐COF (square‐symbol), and TAPB‐PDA‐COF (triangle‐symbol). Uptakes were measured at A–C) 200 K and B–D) 298 K at 1 bar.

To further confirm the porous character of our COF‐membranes, we also measured the adsorption capacity of CH_4_ and CO_2_ at several temperatures for both COF‐aerogels and COF‐membranes (**Figure** **3**). Notably, COF‐aerogels showed high adsorption of both CO_2_ and CH_4_: CO_2_ adsorptions (e.g., 22.6 mmol g^−1^ at 200 K for TAPB‐PDA‐AGCOF) are in the range of other COFs with great CO_2_ adsorption capacity (Figure S29, Supporting Information); and CH_4_ adsorptions (e.g., 3.8 mmol g^−1^ for TAPB‐BTCA‐AGCOF) are among the highest described for 2D‐COFs at low‐pressures (1 bar) (Figure S30, Supporting Information). Compared to their corresponding aerogels, the maximum adsorption capacities of the membranes for CO_2_ and CH_4_ (200 K, 100 kPa) decreased in the range of 40–50% for TAPB‐BTCA‐MCOF, 40–44% for PPDA‐BTCA‐MCOF, and 63–76% for TAPB‐PDA‐MCOF. Thus, the maximum CO_2_ uptake for each membrane was 8.1 mmol g^−1^ at 200 K (1.2 mmol g^−1^ at 298 K), 6.6 mmol g^−1^ at 200 K (1.0 mmol g^−1^ at 298 K), and 5.4 mmol g^−1^ at 200 K (0.6 mmol g^−1^ at 298 K) for TAPB‐BTCA‐MCOF, PPDA‐BTCA‐MCOF, and TAPB‐PDA‐MCOF, respectively. For CH_4_, the maximum uptakes were 1.8 mmol g^−1^ at 200 K (0.3 mmol g^−1^ at 298 K) for TAPB‐BTCA‐MCOF; 1.7 mmol g^−1^ at 200 K (0.3 mmol g^−1^ at 298 K) for PPDA‐BTCA‐MCOF; and 1.0 mmol g^−1^ at 200 K (0.1 mmol g^−1^ at 298 K) for TAPB‐PDA‐MCOF. Together with PXRD data, these results confirm that, although the compression of COF‐aerogels reduces their gas adsorption capacity, the resulting COF‐membranes are still porous.

**Figure 4:**
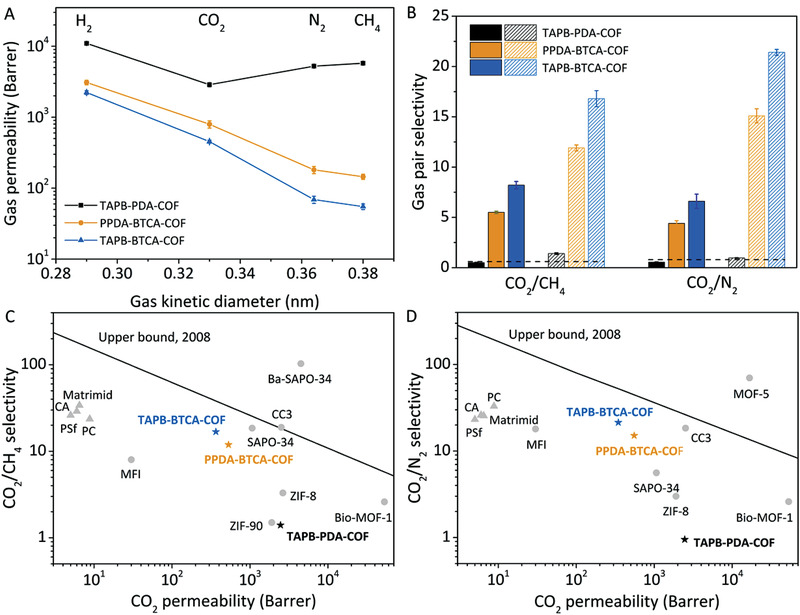
A) Gas permeation properties of TAPB‐BTCA‐MCOF, PPDA‐BTCA‐MCOF, and TAPB‐PDA‐MCOF obtained by single gas measurements at 298 K under a transmembrane pressure of 1 bar. B) Ideal gas pair selectivity (solid) and mixed gas pair selectivity (or separation factor, empty) of TAPB‐BTCA‐MCOF, PPDA‐BTCA‐MCOF, and TAPB‐PDA‐MCOF. C) CO_2_/CH_4_ and (D) CO_2_/N_2_ upper bound plots for all the three COF‐membranes.

Once demonstrated that imine‐based COF‐membranes synthesized via compression are porous and show good mechanical properties, we evaluated their gas permeation properties by single and mixed gas measurements at 298 K under a transmembrane pressure of 1 bar (**Figure** **4**). A Knudsen diffusion phenomenon was observed in TAPB‐PDA‐MCOF during the single gas tests (Figure 4A). This behavior agrees with the fact that TAPB‐PDA‐MCOF is a mesoporous membrane with a pore size of 3.2 nm, which is much larger than the diameters of gases. For instance, this membrane exhibited an ideal H_2_/CO_2_ selectivity of 3.8, close to the H_2_/CO_2_ selectivity of 4.7 calculated from the Knudsen diffusion theory. In the case of TAPB‐BTCA‐MCOF and PPDA‐BTCA‐MCOF, these factors were higher than the corresponding ideal selectivity for separating CO_2_/CH_4_ and CO_2_/N_2_. For example, while the ideal CO_2_/CH_4_ selectivity is 8.2, the CO_2_/CH_4_ separation factor for TAPB‐BTCA‐MCOF reached 16.8. These results could be rationalized by the coadsorption process in the mixed gas separation process or by the adsorption of water that can enhance the CO_2_/CH_4_ separation factor, as previously reported for related COFs.^[^ ^29^ ^]^

These observations agree with the presence of imine groups into the COF cavities since it is known that the presence of some heteroatoms may enhance adsorption capacities of COFs.^[^ ^30^ ^]^ A separation mechanism based on an adsorption‐diffusion process is expected for imine‐based COFs, in which imine groups located at the COFs pores favor the CO_2_ adsorption due to weak interactions between the nitrogen of imine bond (Lewis bases) and CO_2_ (Lewis acid). In contrast, these interactions are missing with the CH_4_ molecules. Thus, when CO_2_ is adsorbed into the COF pore induces a coadsorption of additional CO_2_ molecules from the gas mixture giving rise to a pore size reduction that enables the separation of CO_2_ versus CH_4_. Therefore, the sorption and diffusion of CH_4_ molecules into the COF‐membranes are retarded.^[^ ^29^ ^]^

Additionally, it has been already reported that the presence of moisture in the COF pores can enhance the CO_2_/CH_4_ separation factor due to water adsorption,^[^ ^29^ ^]^ in which the presence of water in the membranes reduces gas permeabilities due to lower gas diffusivities and solubilities (i.e., higher CO_2_ sorption in water overwhelms the reduced CO_2_ diffusivity due to the strong affinity between CO_2_ molecules and water molecules in terms of permeability.^[^ ^31^ ^]^

It is worth noting that permeability is the product of solubility and diffusivity. Therefore, the CH_4_ permeability decreases from 55 Barrer in the single gas test to 21 Barrer in the mixed gas test. We then plotted the separation performance of TAPB‐BTCA‐MCOF, PPDA‐BTCA‐MCOF, and TAPB‐PDA‐MCOF and compared them with other membranes on the Robeson upper bound plots (Figure 4C). Therefore, these new COF‐membranes show higher CO_2_ permeability than commercial membranes (such as CA, PSf, PC, and Matrimid) and separation performance close to the 2008 upper bound limits and better than reported for some MOFs (ZIF‐8 and Bio‐MOF‐1) and Zeolites (MFI and SAPO‐34) (Tables S8 and S9, Supporting Information). With this data, we tested the working performance of the best membrane: TAPB‐BTCA‐MCOF. To this end, we studied the pressure influence (transmembrane pressure from 1 to 6 bar) on the separation performance of TAPB‐BTCA‐MCOF in mixed gas tests (Figure S31A,B, Supporting Information). With increasing the transmembrane pressure, the gas permeability rapidly increased, and the gas pair selectivity quickly decreased, which indicated that the defective sites in the membrane at higher pressures increased. These defects may arise from intercrystalline boundaries present in TAPB‐BTCA‐MCOF.

Next, we evaluated the separation performance of TAPB‐BTCA‐MCOF under different temperatures for two different mixed gas tests (Figure S32C,D, Supporting Information). We found that the gas permeability increased by increasing the temperature due to the fast diffusion rates of gases at higher temperatures. From data collected in Figure S32C,D (Supporting Information), we calculated activation energies for TAPB‐BTCA‐MCOF of 9.39 (CO_2_) and 13.88 (CH_4_) kJ mol^−1^ for the separation of CO_2_/CH_4_, and 9.31 (CO_2_) and 14.13 (N_2_) kJ mol^−1^ for the separation of CO_2_/N_2_ confirming a stronger interaction of TAPB‐BTCA‐MCOF with CO_2_ than with CH_4_ and N_2_.

Finally, we evaluated the long‐term stability of TAPB‐BTCA‐MCOF for the separation of CO_2_/CH_4_ mixtures (Figure S33, Supporting Information). We introduced moisture (relative humidity: 85%) during the test, and the stabilized membrane showed a rather steady separation performance. The reduction of gas permeability is due to the partial block of pores by water under humid conditions.^[^ ^32^ ^]^

## 3 Conclusion

In summary, we report a simple compression method for the fabrication of centimeter‐scale imine‐based COF‐membranes from COF‐aerogel. Applying pressure on COF‐aerogels allows their transformation into free‐standing COF‐membranes that preserve their original crystallinity and are porous. Moreover, the fabricated free‐standing COF‐membranes have shown an enhancement in their mechanical properties, i.e., Young modulus and flexibility, enabling them to be used for gas separation processes under pressure conditions. The separation performances of TAPB‐BTCA‐MCOF for the gas mixture CO_2_/CH_4_ or CO_2_/N_2_ are close to the upper bound limits. Therefore, we believe that this novel processing method, once optimized, opens new avenues to produce large‐scale COF‐membranes with controlled size and thickness for different applications, going from nanofiltration to proton membranes.

## 4 Experimental Section

### Materials

Most chemicals and solvents were obtained from Aldrich Chemical Co. and used without further purification. Ethanol (EtOH) and tetrahydrofuran (THF) were dried using standard methods and stored under an inert gas atmosphere.^[^ ^33^ ^]^ TAPB was synthesized as previously reported.^[^ ^22a^ ^]^ BTCA, PDA, and PPDA were commercially available and were used as purchased with no further treatment.

### Synthesis of COF‐Aerogels

The synthesis and characterization of COF‐aerogels were made according to the published procedures described previously by us.^[^ ^21^ ^]^

### Membrane Fabrication

Membranes of TAPB‐BTCA‐AGCOF, PPDA‐BTCA‐AGCOF, and TAPB‐PDA‐AGCOF were prepared by breaking (10 mg) of the COF‐aerogel material into small pieces (size: ≈1–3 mm) in the presence of (5 µL) acetic acid (AcOH). Then, a pressure of 120 MPa was applied, using a uniaxial hydraulic press, for 5 min. Under these conditions, COF‐membranes showed around 13 mm of diameter and a thickness of 50–60 µm (Figure S1A, Supporting Information). For the 20 mm in diameter COF‐membranes (Figure S1B, Supporting Information), they were prepared by breaking (25 mg) of the COF‐aerogel material into small pieces (size: ≈1–3 mm) in the presence of (15 µL) acetic acid (AcOH). Then, a pressure of 120 MPa was applied, using a uniaxial hydraulic press, for 20 min.

In the case of 50 mm in diameter COF‐membranes (Figure S1C, Supporting Information), they were prepared by breaking (150 mg) of the COF‐aerogel material into small pieces (size: ≈1–3 mm) in the presence of (80 µL) acetic acid (AcOH). Then, a pressure of 30 MPa was applied, using a uniaxial hydraulic press, for 60 min.

### Chemical Stability Test

Chemical stability tests were carried out by immersing the COF‐membranes in (1 mL) aqueous solutions of hydrochloric acid (HCl) (12 m), sodium hydroxide (NaOH) (14 m), and common solvents (toluene, dimethylformamide, and hexane) under stirring for 72 h. Then, the samples were washed with water (only for the incubations with HCl and NaOH), tetrahydrofuran (THF), and ethanol (EtOH) and dried under vacuum at 333 K for 24 h. In the case of the acidic and basic treatments, 93% and 98% of the solid material were recovered. 100% of the material was recovered after chemical stability tests with common solvents. In all of these experiments, there was no significant loss of crystallinity.

### Characterizations

PXRD patterns were collected with a Bruker D8 Advance X‐ray diffractometer (Cu K*α* radiation; *λ* = 1.5418 Å) equipped with a Lynxeye detector. Samples were mounted on a flat sample plate. Patterns were collected in the 3.5° < 2*θ* < 35° range with a step size of 0.025° and exposure time of 1.3 s step^−1^. Elemental analyses were obtained using LECO CHNS‐932 elemental analyzer. FT‐IR spectra were recorded in a Perkin Elmer Spectrum 100 with a PIKE Technologies MIRacle Single Reflection Horizontal ATR (attenuated total reflection) accessory with a spectral range of 4000–650 cm^−1^, signals are given in wavenumbers (cm^−1^). N_2_ adsorption isotherms were measured using a Micromeritics ASAP2020 volumetric instrument under static adsorption conditions. Before measurement, samples were heated at 323 K overnight and outgassed to 10^−6^ Torr. Brunauer–Emmet–Teller (BET) and Langmuir analyses were carried out to determine the total specific surface areas from the N_2_ isotherms at 77 K. The pore size distribution (PSD) was determined from N_2_ adsorption isotherms at 77 K using nonlocal DFT for a model with cylindrical pores present in software MicroActive Version 4.06 of Micromeritics. Solid‐State ^13^C NMR spectra were carried out on a Bruker AV 400 WB Spectrometer. Carbon chemical shifts are expressed in parts per million (*δ* scale). SEM studies were performed on a JSM‐7600 equipped with an OXFORD X‐Max XEDS, operating at an accelerating voltage of 10 kV. Samples were previously coated with gold in a sputter Quorum Q150T‐S. The samples were prepared by gluing, in perpendicular, a piece of COF‐membrane in carbon tape. TGA were run on a Thermobalance TGA Q‐500 thermal gravimetric analyzer with samples held in an aluminum pan under a nitrogen atmosphere. The samples were heated at 10 K min^−1^ within a temperature range of 25–1000 °C.

### Mechanical Properties and Topography Characterization by AFM Imaging

AFM measurements were carried out using a Cervantes Full mode AFM from Nanotec Electronica SL. WSxM software (www.wsxmsolutions.com) was employed both for data acquisition and image processing.^[^ ^25^ ^]^ Topography images in amplitude modulation mode using a hemispherical cone‐shaped tip with a radius of 120 nm from Team Nanotec, with a resonance frequency of 348 kHz and a spring constant of 46 N m^−1^, calibrated using Sader's method were acquired.^[^ ^34^ ^]^ The same tip was used to carry out the nanoindentation experiments. This consisted in acquiring a force versus distance curve in each pixel of the image to determine Young's modulus of the membranes by fitting the corresponding indentation curves to the Hertz model.^[^ ^26^ ^]^ Further measurements were repeated using a different tip with a nominal radius of 250 nm, obtaining similar results, proving the robustness of the measurements. After acquiring a force versus distance curve in each pixel, the indentation curve is obtained by using the spring constant of the cantilever to subtract the effect of the cantilever deflection from the aforementioned curve, leaving just the indentation length of the tip into the sample. With these indentation curves it is possible to obtain the Young's Modulus by fitting the model of Hertzian Contact for a spherical indenter: *F* = 4/3 × *E* × *R* ^1/2^ × *d* ^3/2^, where *F* is the normal force, *R* is the tip radius, *d* is the indentation length, and *E* is Young's Modulus obtained from the fit.

### Membrane Separation Performance

It was determined by single gases or gas mixtures (50:50 vol%) in a Wicke–Kallenbach gas permeation setup. The volumetric flow rates of the feed gas and sweep gas (argon) were maintained at 50 and 50 mL min^−1^, respectively. The gas compositions at the permeate side were analyzed using a gas chromatograph (Shimadzu GC‐2014). All membranes were prepared at least three times to verify their reproducibility. The gas permeability (*P*_i_, barrer, 1 barrer = 10^−10^ cm^3^ (STP) cm^−2^ s^−1^ cm Hg^−1^) is defined as *P_i_* = (*Q_i_l*)/(*A*Δ*P_i_*), where *Q_i_* is the volume flow rate of gases (cm^3^ s^−1^), *l* is the membrane thickness (µm), *A* is the effective area of the membrane (cm^2^), and Δ*P_i_* is the partial pressure difference across the membrane (cm Hg). The mixed gas selectivity (*α*_*ij*_) is defined as: *α*_ij_ = (*y_i_*/*y_j_*)/(*x_i_*/*x_j_*), where *x_i_*/*x_j_* and *y_i_*/*y_j_* represent the molar fraction of *i*/*j* in the feed and permeate side, respectively. When the membranes were tested under moisture, the feed gas was humidified by passing through a water bottle at room temperature (relative humidity: ≈85%).

## Conflict of Interest

The authors declare no conflict of interest.

## Supporting information

Supporting Information

## Data Availability

The data that support the findings of this study are available from the corresponding author upon reasonable request.
