# Supplementary material for: Ultralarge Free‐Standing Imine‐Based Covalent Organic Framework Membranes Fabricated via Compression
Source: Adv Sci (Weinh). 2022 Jan 17;9(7):2104643. doi: 10.1002/advs.202104643 (PMC8895050; doi:10.1002/advs.202104643)
Supplement: Supplementary file 1 — Supporting Information [file ADVS-9-2104643-s001.pdf]

ADVANCED SCIENCE
Open Access

Supporting Information

for *Adv. Sci.*, DOI: 10.1002/advs.202104643

Ultra-Large Free-Standing Imine-based Covalent Organic Framework Membranes Fabricated via Compression

*Jesús Á. Martín-Illán, José Antonio Suárez, Julio Gómez-Herrero, Pablo Ares, Daniel Gallego-Fuente, Youdong Cheng, Dan Zhao, Daniel Maspoch and Félix Zamora**

Supporting Information

Ultra-Large Free-Standing Imine-based Covalent Organic Framework Membranes Fabricated via Compression

*Jesús Á. Martín-Illán, José Antonio Suárez, Julio Gómez-Herrero, Pablo Ares, Daniel Gallego-Fuente, Youdong Cheng, Dan Zhao, Daniel Maspoch and Félix Zamora**

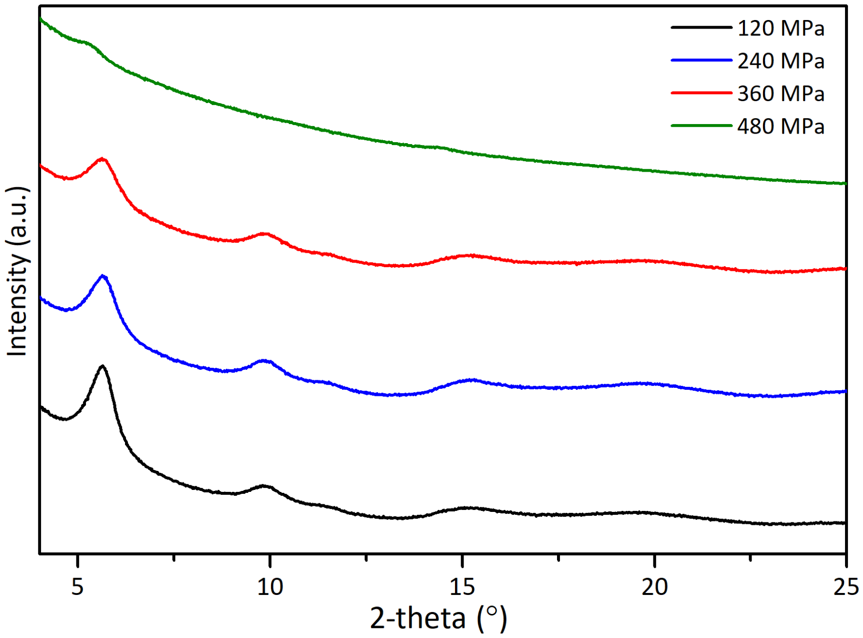

Figure S1. PXRD patterns of TAPB-BTCA-MCOFs at different pressures in *perpendicular* mode.

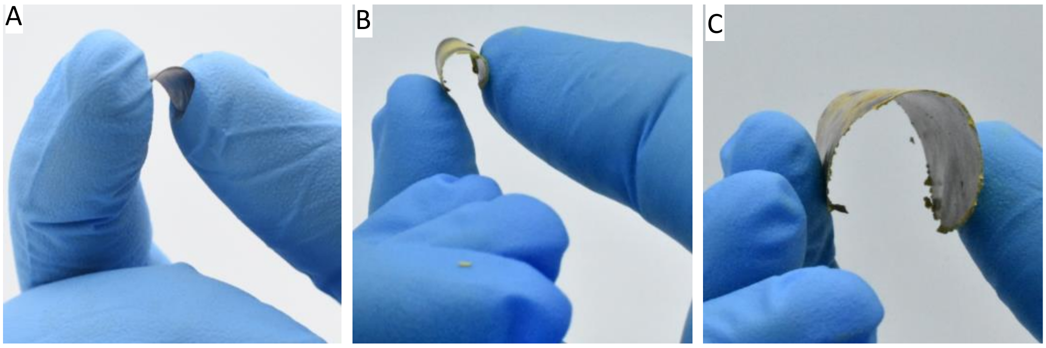

Figure S2. Photographs highlighting the flexible COF-membrane. (A) 1.3 cm (B) 2 cm and (C) 5 cm of diameter.

Table S1. Experimental elemental analysis data of COF aerogels (AG) and COF-membranes (M).

Sample	%C	%H	%N
TAPB-BTCA-AGCOF	81.83	4.96	7.79
TAPB-BTCA-MCOF	79.74	5.03	8.26
PPDA-BTCA-AGCOF	74.20	4.91	14.03
PPDA-BTCA-MCOF	69.97	5.42	10.63
TAPB-PDA-AGCOF	83.49	5.19	7.87
TAPB-PDA-MCOF	80.0	5.28	7.58

Calculated Elemental Analysis:

- **TAPB-BTCA-AGCOF. $C_{33}H_{21}N_3(H_2O)_{1.5}$: C, 81.16%; H, 4.99%; N, 8.6%.**
- **TAPB-BTCA-MCOF. $C_{33}H_{21}N_3(H_2O)$ (CH_3COOH): C, 78.19 %; H, 5.06%; N, 7.82 %. (Figure S9).**
- **PPDA-BTCA-AGCOF. $C_{36}H_{24}N_6(H_2O)_{2.5}$: C, 73.94%; H, 4.98%; N, 14.37 %.**
- **PPDA-BTCA-MCOF. $C_{36}H_{24}N_6(H_2O)_{2.5}$ (CH_3COOH): C, 70.68%; H, 5.15%; N, 13.02 %. (Figure S10).**
- **TAPB-PDA-AGCOF. $C_{72}H_{48}N_6(H_2O)_{1.5}$: C, 81.16%; H, 4.99%; N, 8.6 %.**
- **TAPB-PDA-MCOF. $C_{72}H_{48}N_6(H_2O)_3(CH_3COOH)$: C, 79.98%; H, 5.26%; N, 7.56 %. (Figure S11).**

Table S2. Physical features of the COF aerogels and membranes.

Sample	Mass [g]	Shape	H [mm]	D [mm]	L1, L2 [mm]	V [cm^3]	ρ [g·cm^{-3}]
TAPB-BTCA-AGCOF	0.057	cylinder	10.0	18.6	--	2.717	0.0209
TAPB-BTCA-MCOF	0.011	disk	0.055	13		0.0073	1.01
PPDA-BTCA-AGCOF	0.150	cylinder	23.0	20.0	--	7.226	0.0208
PPDA-BTCA-MCOF	0.010	Disk	0.054	13		0.0071	1.1
TAPB-PDA-AGCOF	0.025	Prism	10.7	--	15.0, 9.0	1.444	0.0173
TAPB-PDA-MCOF	0.012	cylinder	0.057	13		0.0075	1.05

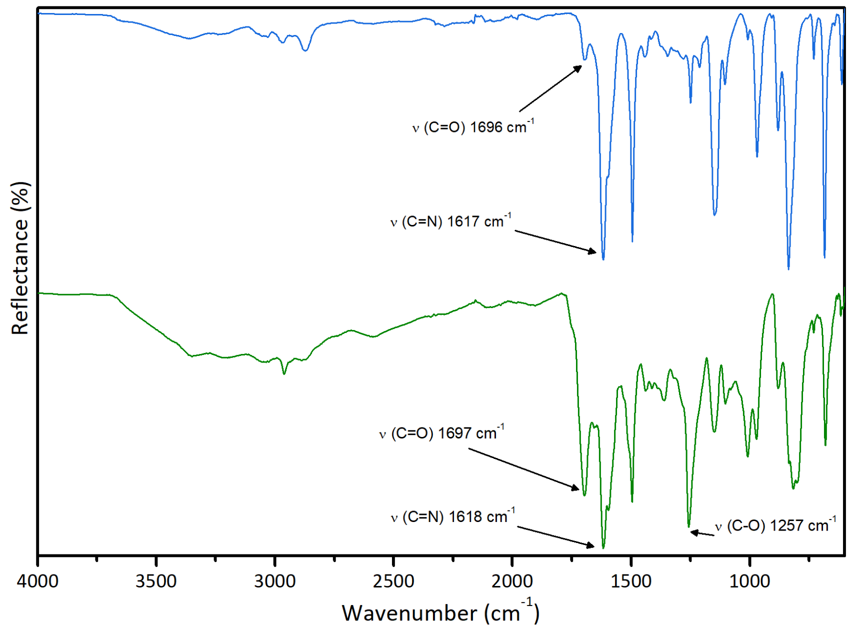

Figure S3. ATR-FT-IR spectra of TAPB-BTCA-AGCOF (blue-line) and TAPB-BTCA-MCOF (green-line).

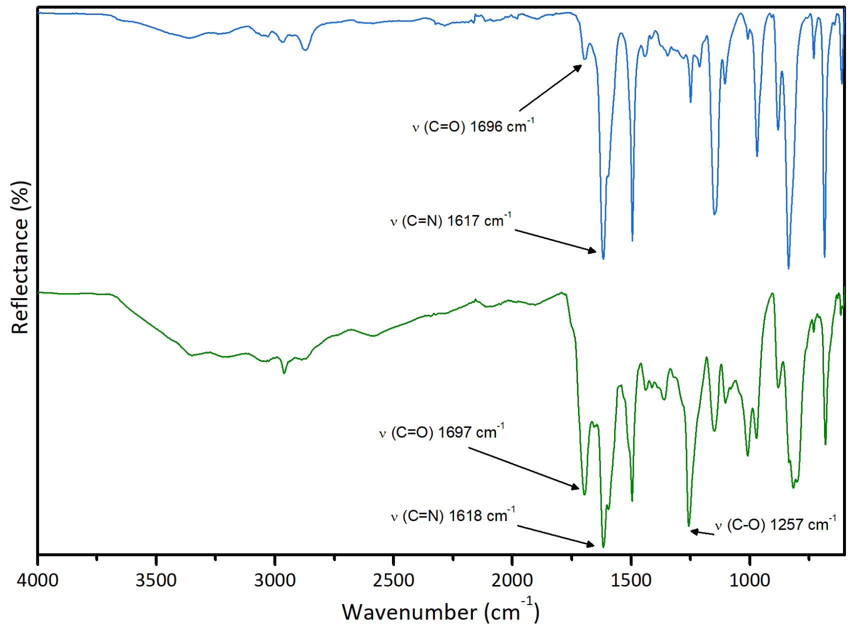

Figure S4. ATR-FT-IR spectra of PPDA-BTCA-AGCOF (blue-line) and PPDA-BTCA-MCOF (green-line).

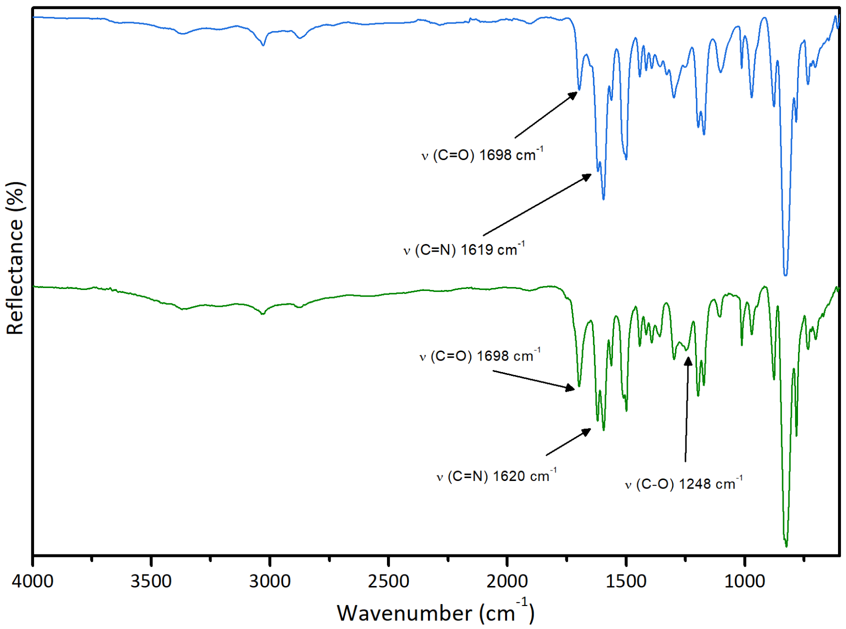

Figure S5. ATR-FT-IR spectra of TAPB-PDA-AGCOF (blue-line) and TAPB-PDA-MCOF (green-line).

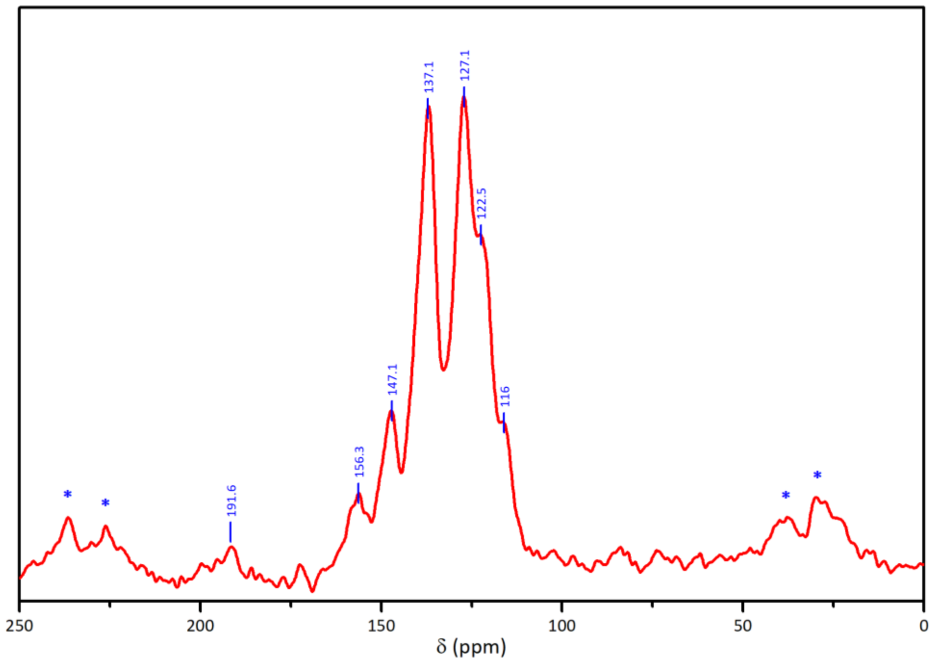

Figure S6. Solid state ^{13}C NMR spectrum of TAPB-BTCA-MCOF.

Table S3. Peaks assignment of solid state ^{13}C NMR spectrum of TAPB-BTCA-MCOF.

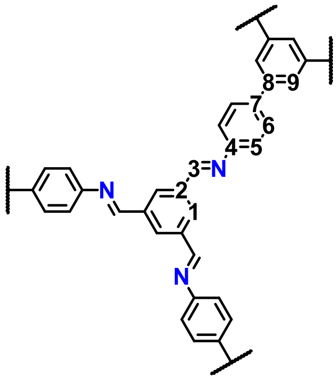

Assignment	Signal [ppm]
1,2	137.1
3	156.3
4	147.1
5	116.0
6,7	127.1
8	137.1
9	122.5

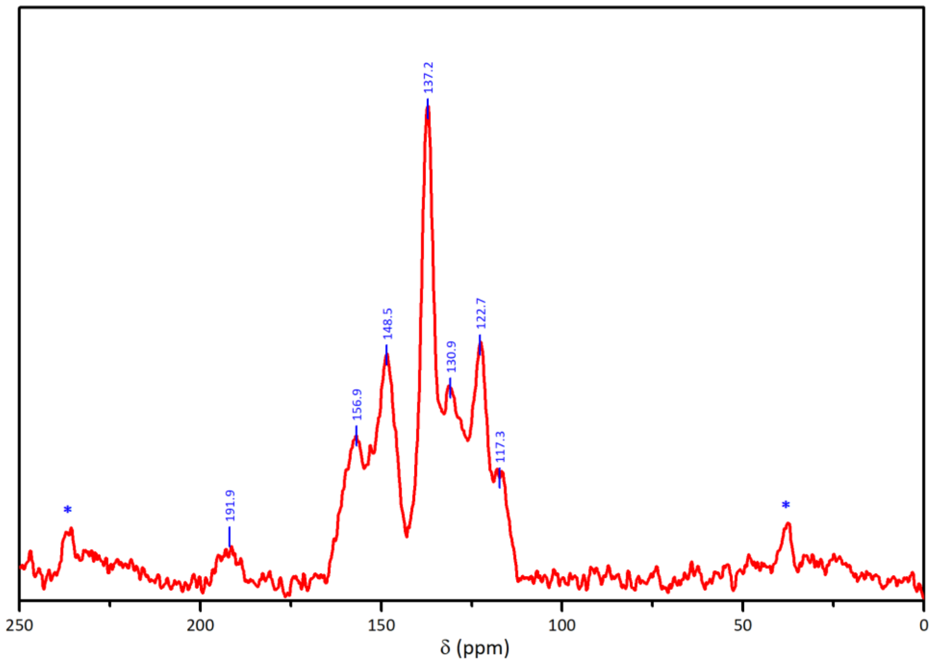

Figure S7. Solid-state ^{13}C CP-MAS NMR spectrum of PPDA-BTCA-MCOF.

Table S4. Peaks assignment of solid state ^{13}C NMR spectrum of PPDA-BTCA-MCOF.

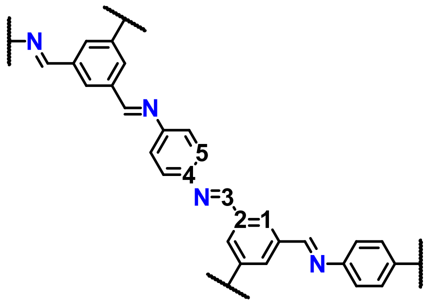

Assignment	Signal [ppm]
1	122.7
2	130.9
3	156.9
4	148.5
5	137.2

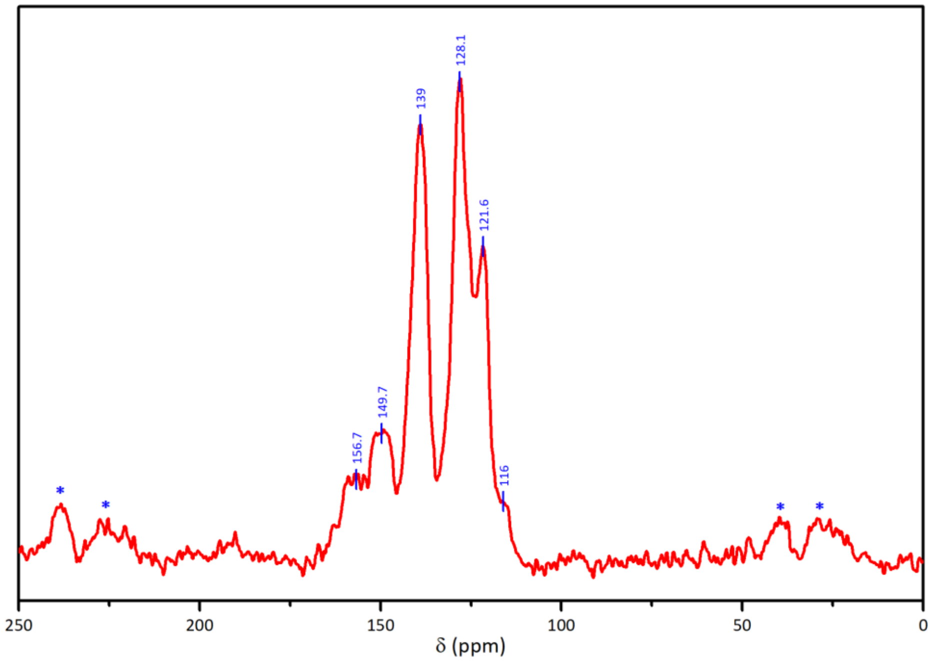

Figure S8. Solid-state ^{13}C CP-MAS NMR spectrum for TAPB-PDA-MCOF.

Table S5. Peaks assignment of solid state ^{13}C NMR spectrum of TAPB-PDA-MCOF.

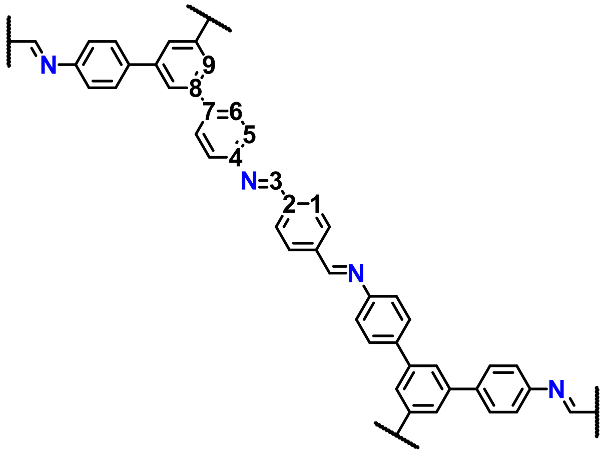

Assignment	Signal [ppm]
1,2	139.0
3	156.7
4	149.7
5	121.6
6,7	128.1
8	139.0
9	121.6

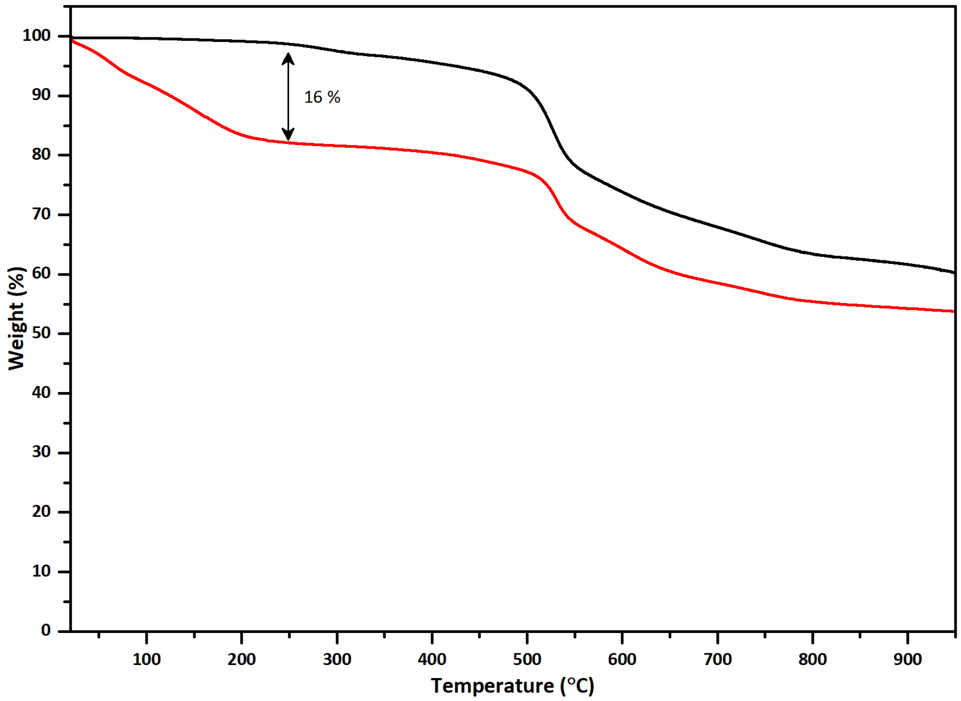

Figure S9. TGA traced for TAPB-BTCA-AGCOF (black) and TAPB-BTCA-MCOF (red). 16 % volatile elements.

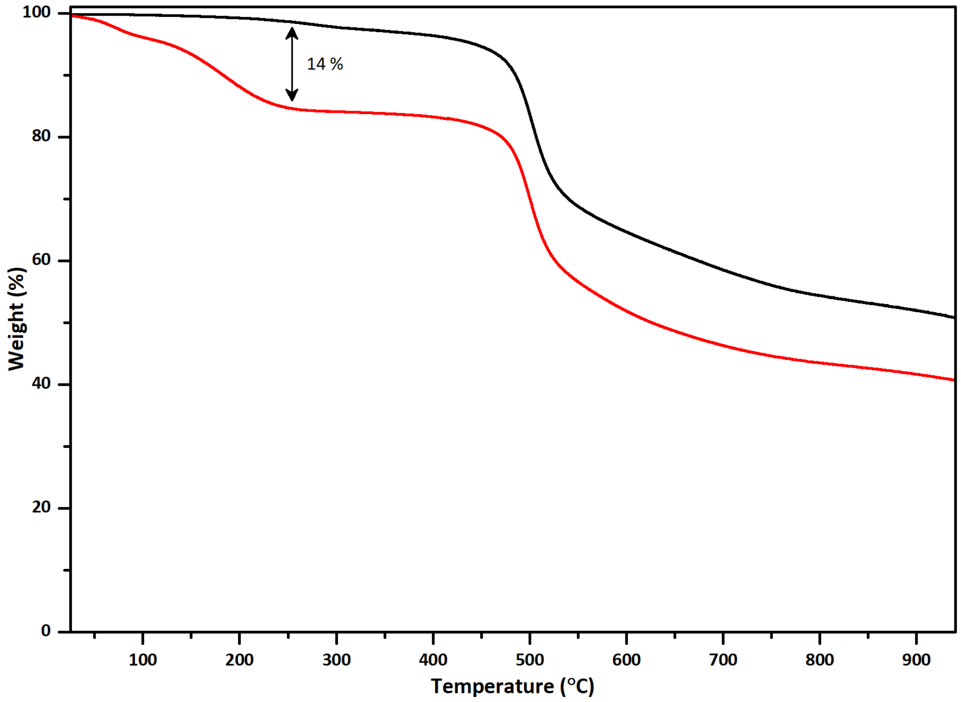

Figure S10. TGA traced for PPDA-BTCA-AGCOF (black) and PPDA-BTCA-MCOF (red). 14 % volatile elements.

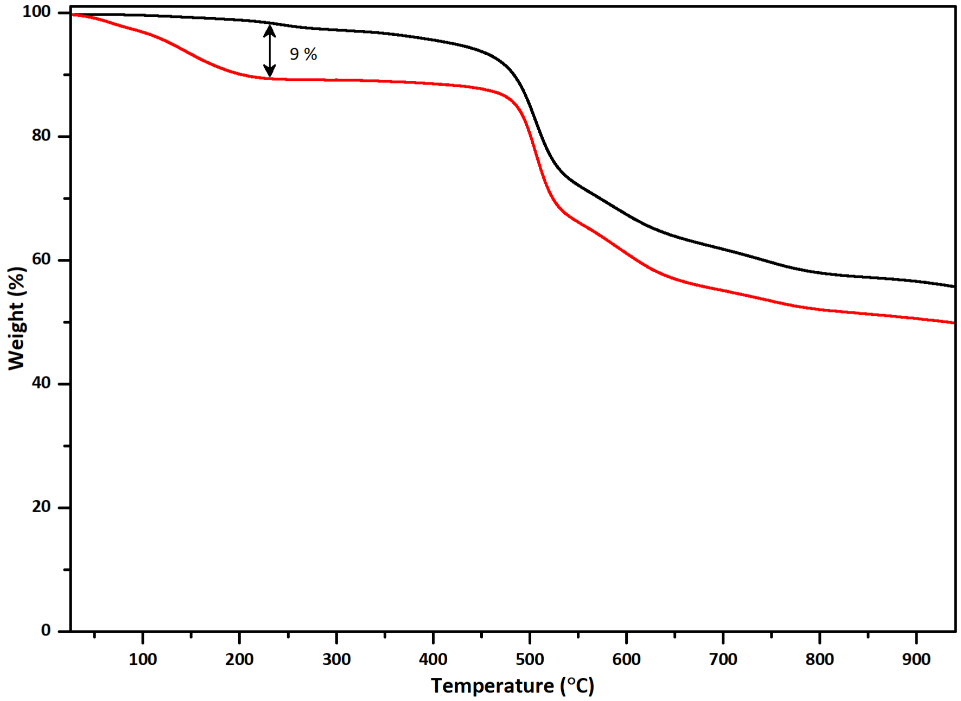

Figure S11. TGA traced for TAPB-PDA-AGCOF (black) and TAPB-PDA-MCOF (red). 9 % volatile elements.

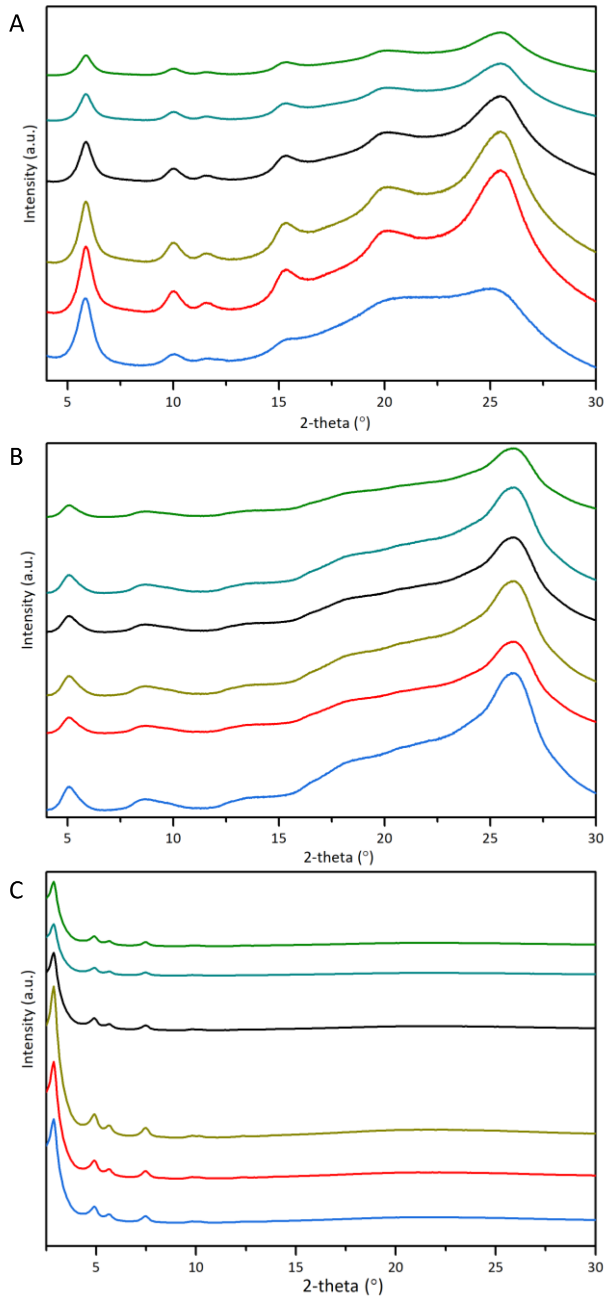

Figure S12. PXRD patterns of (A) TAPB-BTCA-MCOF, (B) PPDA-BTCA-MCOF and (C) TAPB-PDA-MCOF before (blue) and after treatment with toluene (red), hexane (dark-yellow), dimethylformamide (black), 14 M NaOH (dark cyan) and 12 M HCl (green).

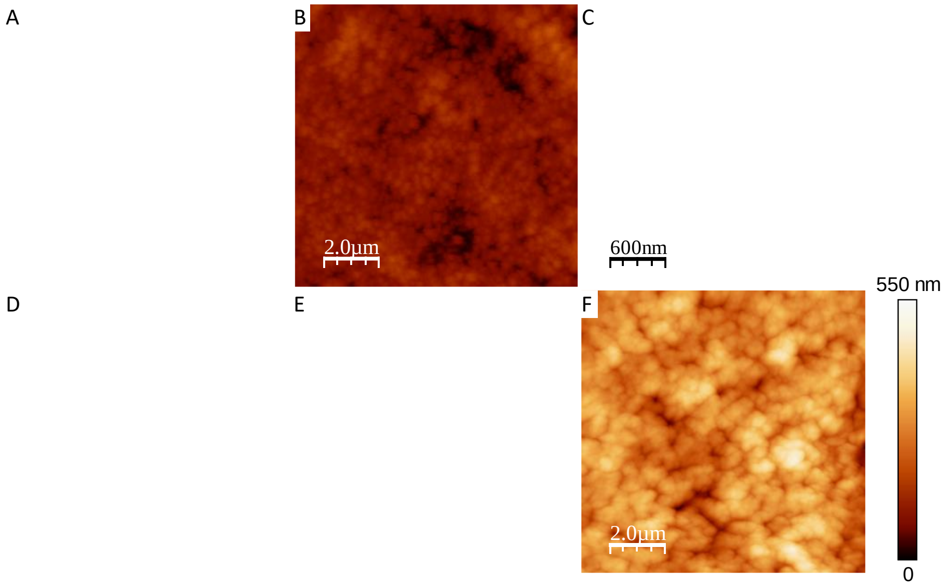

Figure S13. AFM Topography images of TAPB-BTCA-MCOF (A-D), PPDA-BTCA-MCOF (B-E) and TAPB-PDA-MCOF (C-F) membranes. A, B and C without AcOH and D, E and F with AcOH.

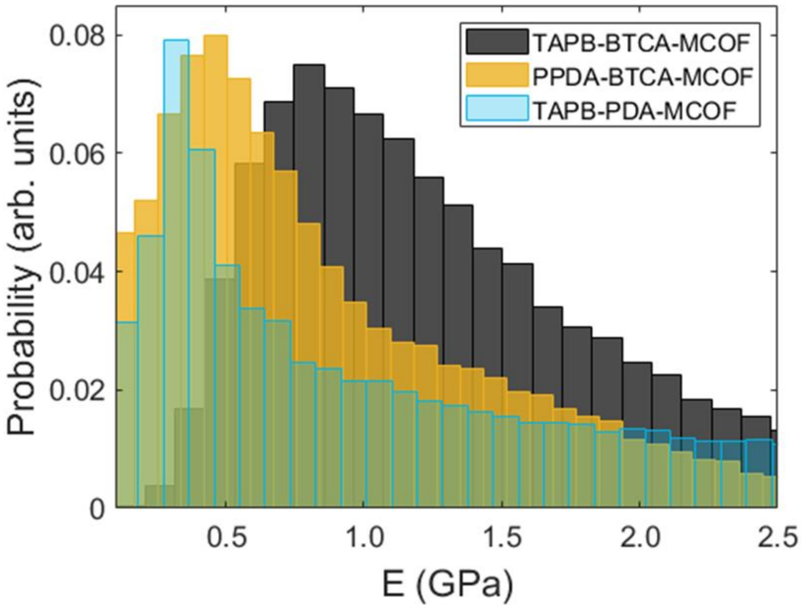

Figure S14. Histograms showing Young's modulus distribution of the membranes with AcOH.

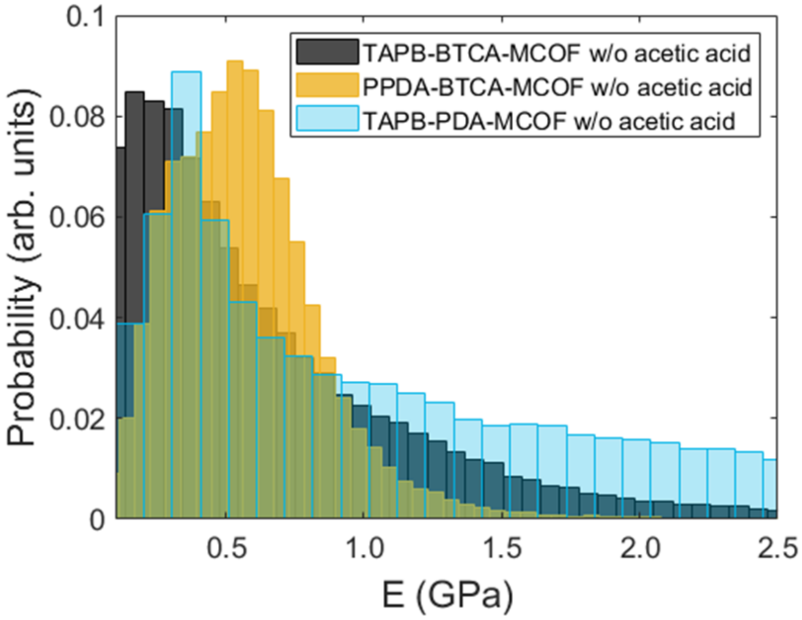

Figure S15. Histograms showing Young's modulus distribution of the membranes without AcOH.

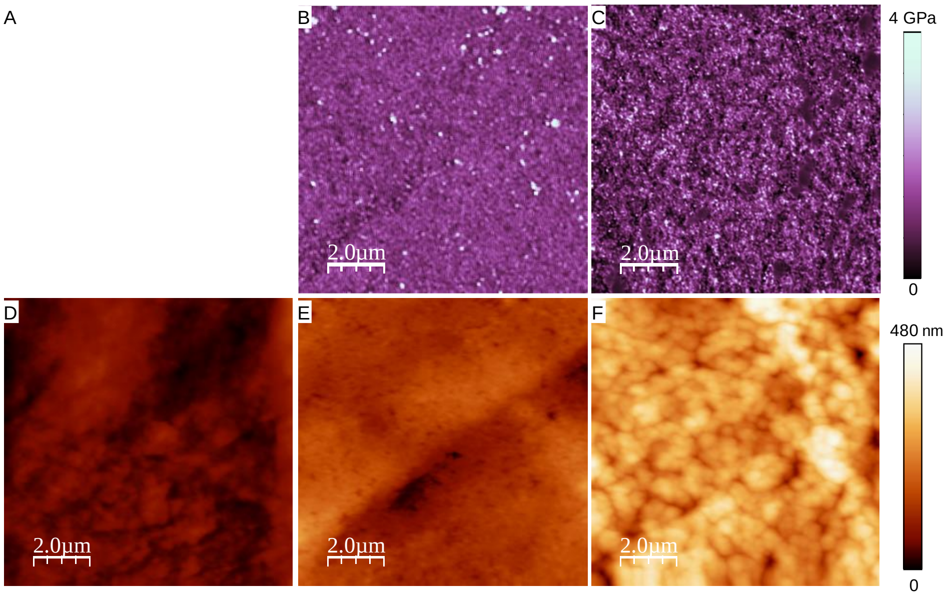

Figure S16. (A, B, C) Representative Young's modulus maps of the TAPB-BTCA-MCOF, PPDA-BTCA-MCOF and TAPB-PDA-MCOF membranes respectively, and the corresponding AFM topographical images of the same areas (D, E, F).

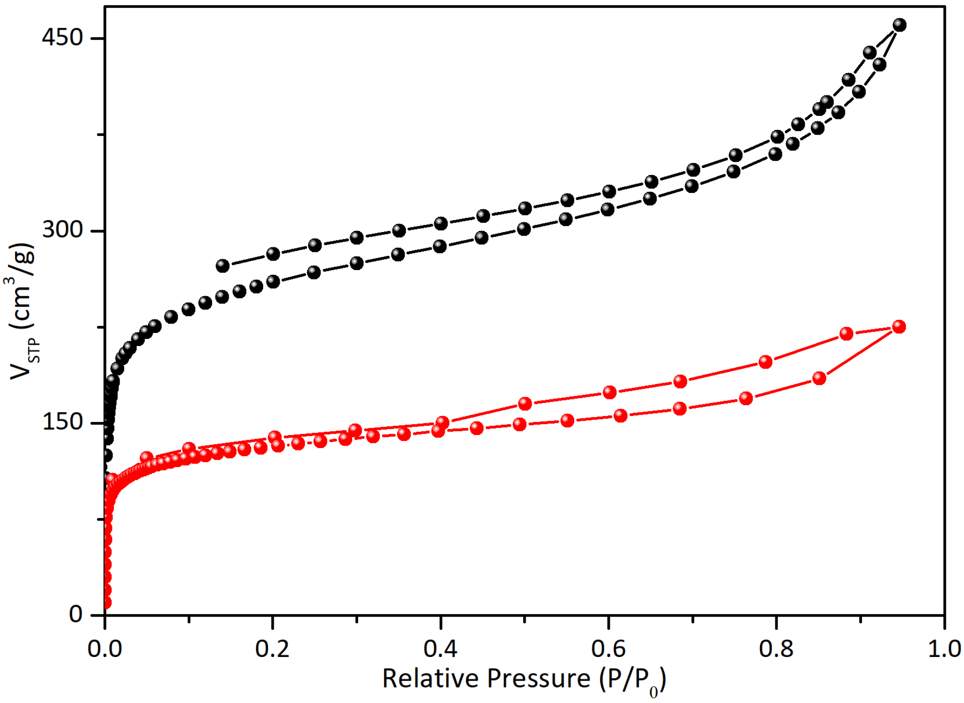

Figure S17. N_2 **adsorption–desorption isotherm of TAPB-BTCA-AGCOF (black line) and TAPB-BTCA-MCOF (red line).**

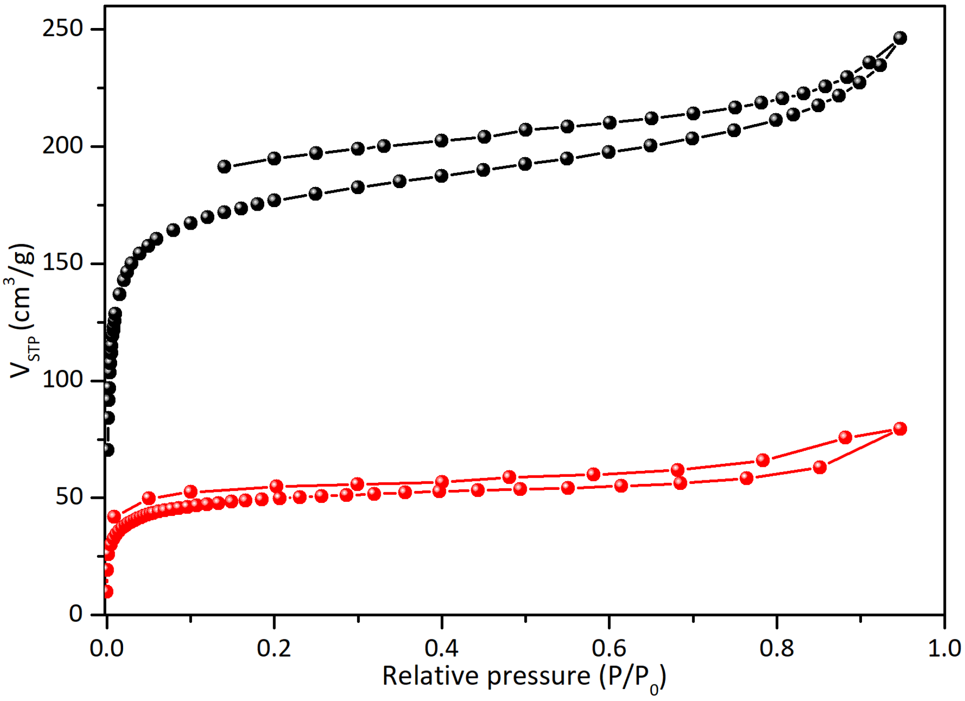

Figure S18. N_2 **adsorption–desorption isotherms of PPDA-BTCA-AGCOF (black line) and PPDA-BTCA-MCOF (red line).**

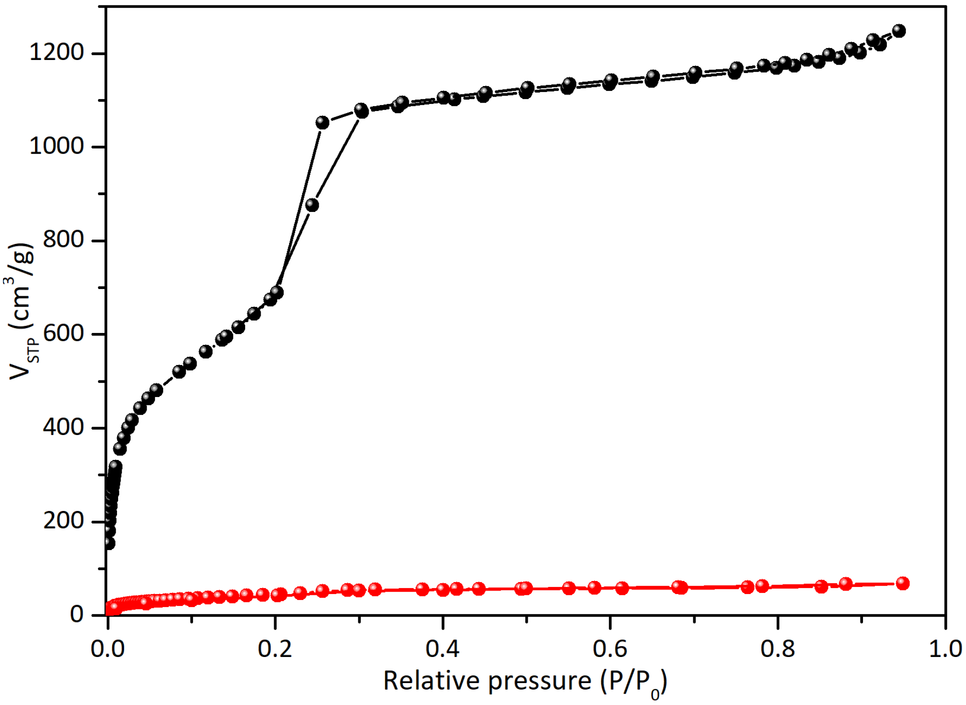

Figure S19. N_2 adsorption–desorption isotherms of TAPB-PDA-AGCOF (black line) and TAPB-PDA-MCOF (red line).

Table S6. BET surface area values for COF-aerogels (AG) and COF-membranes (M).

	Surface area [m^2 g^{-1}]
TAPB-BTCA-AGCOF	1114
TAPB-BTCA-MCOF	247
PPDA-BTCA-AGCOF	677
PPDA-BTCA-MCOF	190
TAPB-PDA-AGCOF	2150
TAPB-PDA-MCOF	170

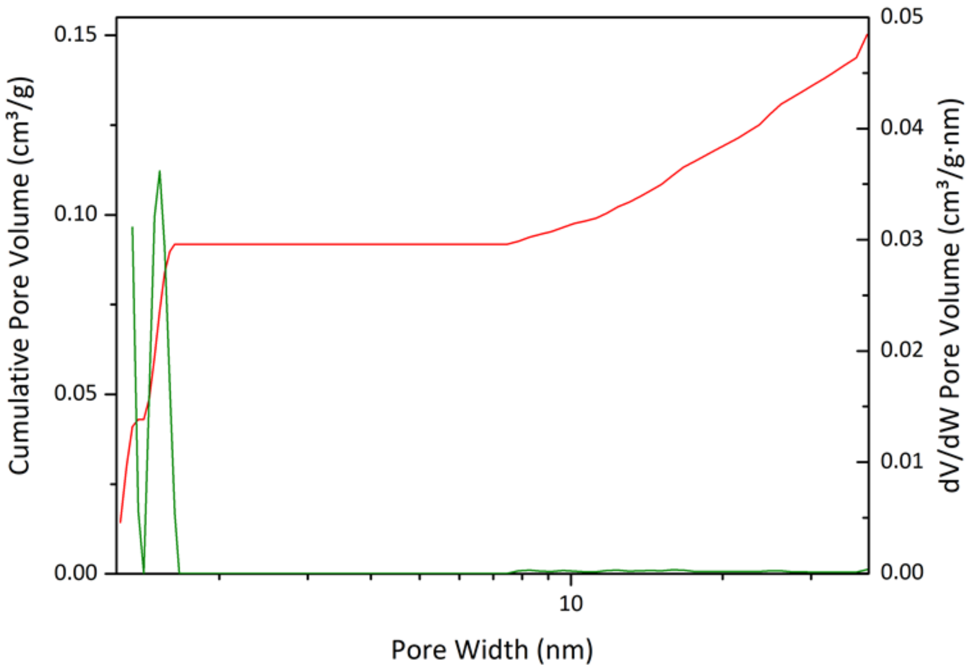

Figure S20. Cumulative and pore size-distribution of TAPB-BTCA-MCOF.

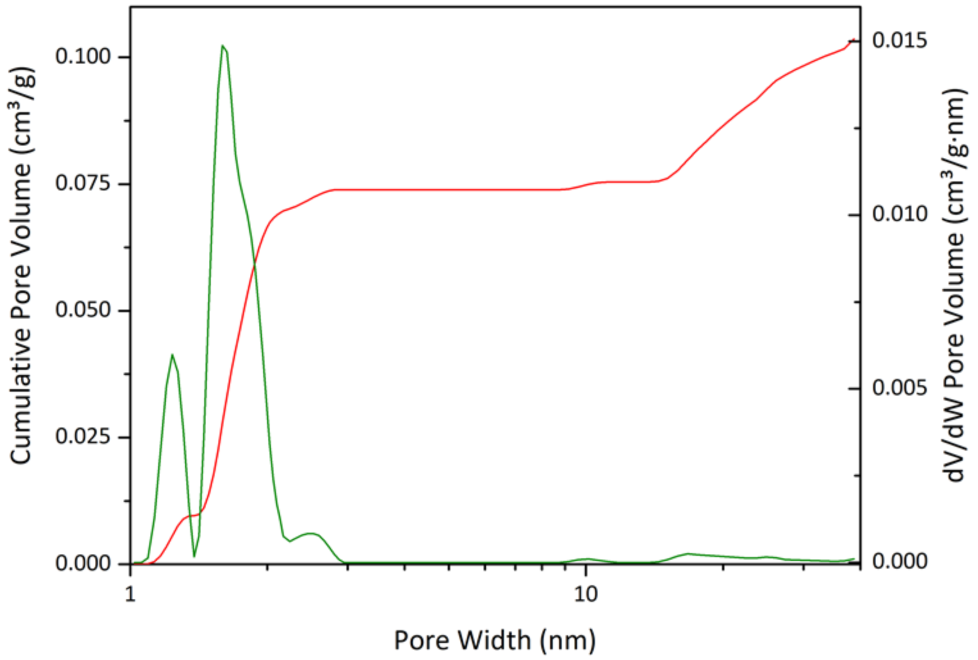

Figure S21. Cumulative and pore size-distribution of PPDA-BTCA-MCOF.

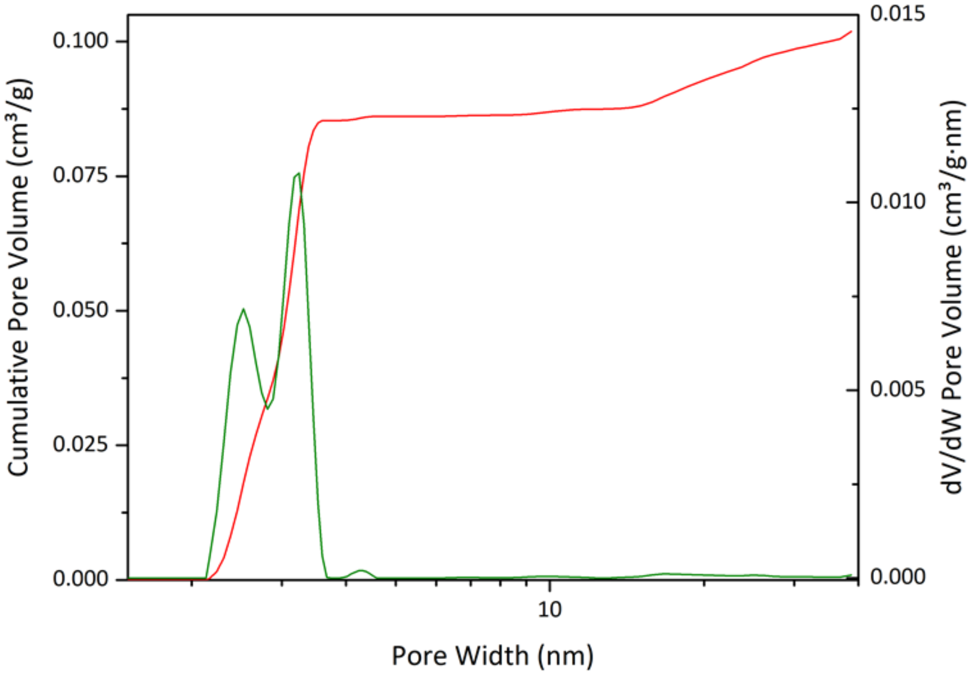

Figure S22. Cumulative and pore size-distribution of TAPB-PDA-MCOF.

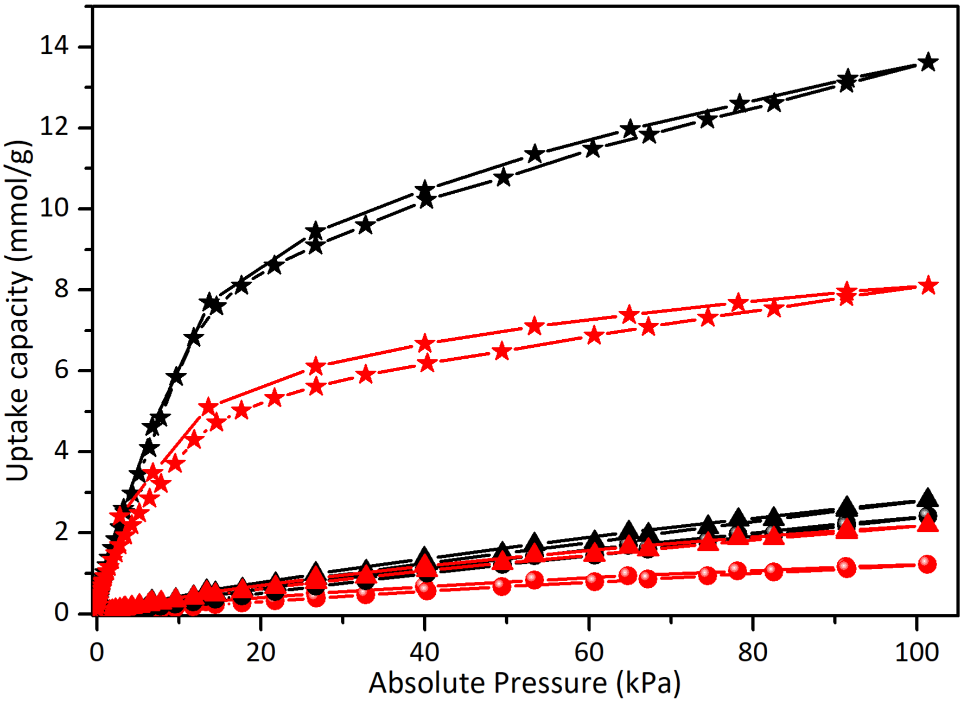

Figure S23. CO_2 uptake capacity isotherms of TAPB-BTCA-AGCOF (black line) and TAPB-BTCA-MCOF (red line). Uptakes were measured at 200 K (star-symbol), 273 K (triangle-symbol) and 298 K (sphere-symbol) at different pressures up to 100 kPa.

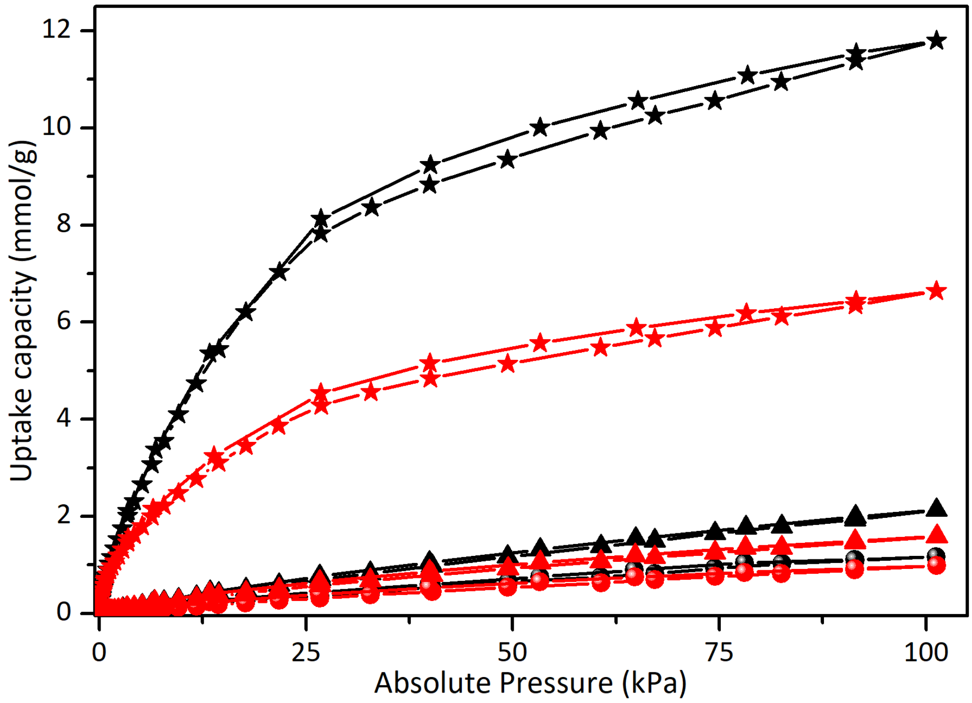

Figure S24. CO_2 **uptake capacity isotherms of PPDA-BTCA-AGCOF (black line) and PPDA-BTCA-MCOF (red line). Uptakes were measured at 200 K (star-symbol), 273 K (triangle-symbol) and 298 K (sphere-symbol) at different pressures up to 100 kPa.**

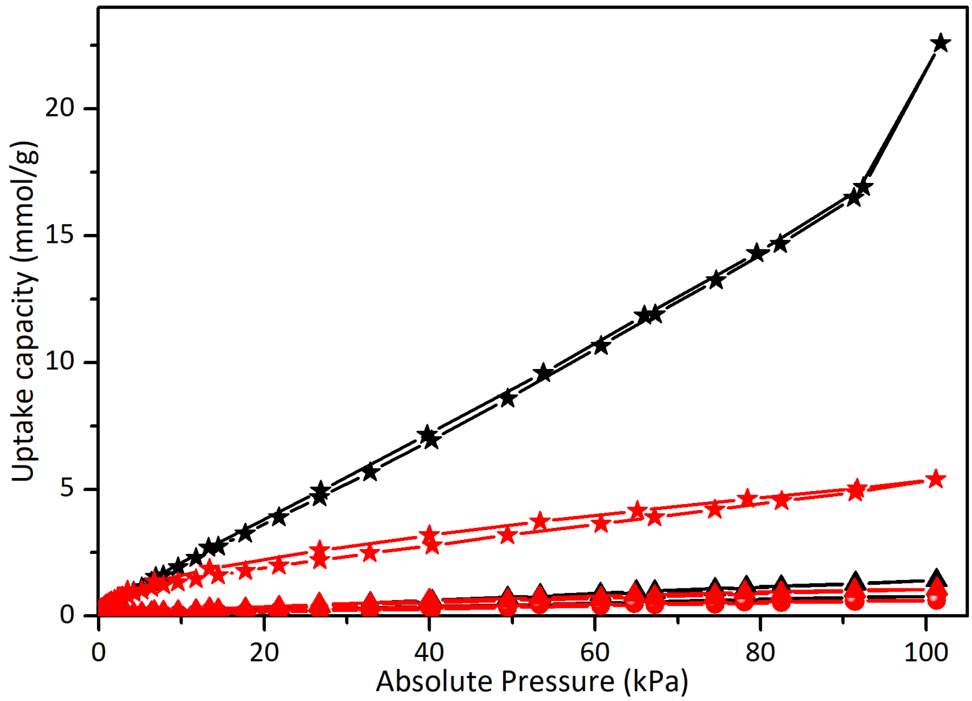

Figure S25. CO_2 **uptake capacity isotherms of TAPB-PDA-AGCOF (black line) and TAPB-PDA-MCOF (red line). Uptakes were measured at 200 K (star-symbol), 273 K (triangle-symbol) and 298 K (sphere-symbol) at different pressures up to 100 kPa.**

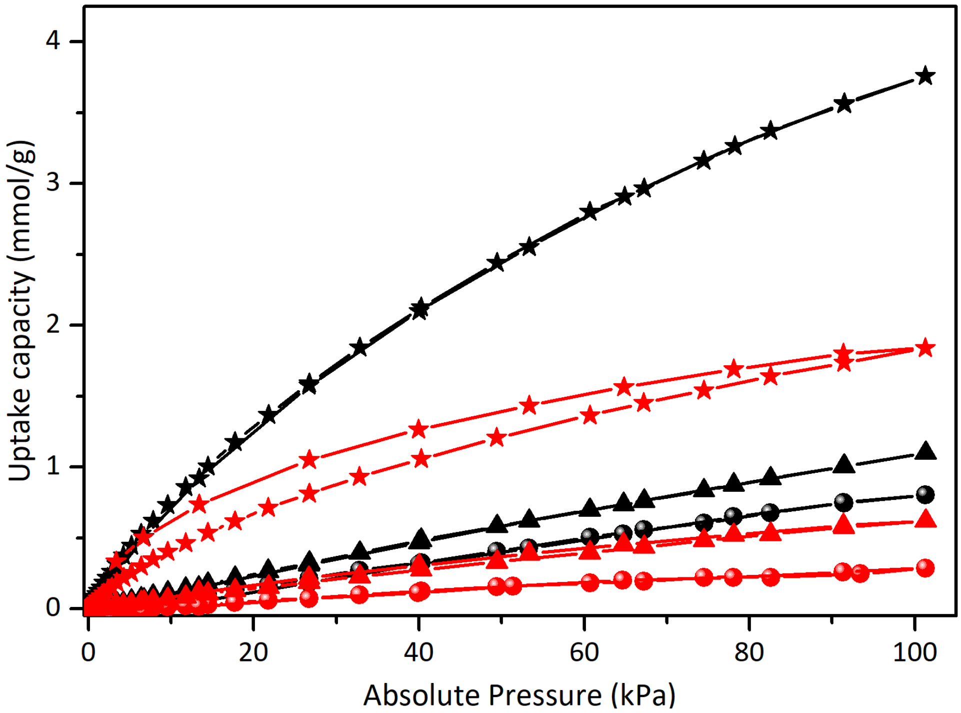

Figure S26. CH_4 uptake capacity isotherms of TAPB-BTCA-AGCOF (black line) and TAPB-BTCA-MCOF (red line). Uptake were measured at 200 K (star-symbol), 273 K (triangle-symbol) and 298 K (sphere-symbol) at different pressures up to 100 kPa.

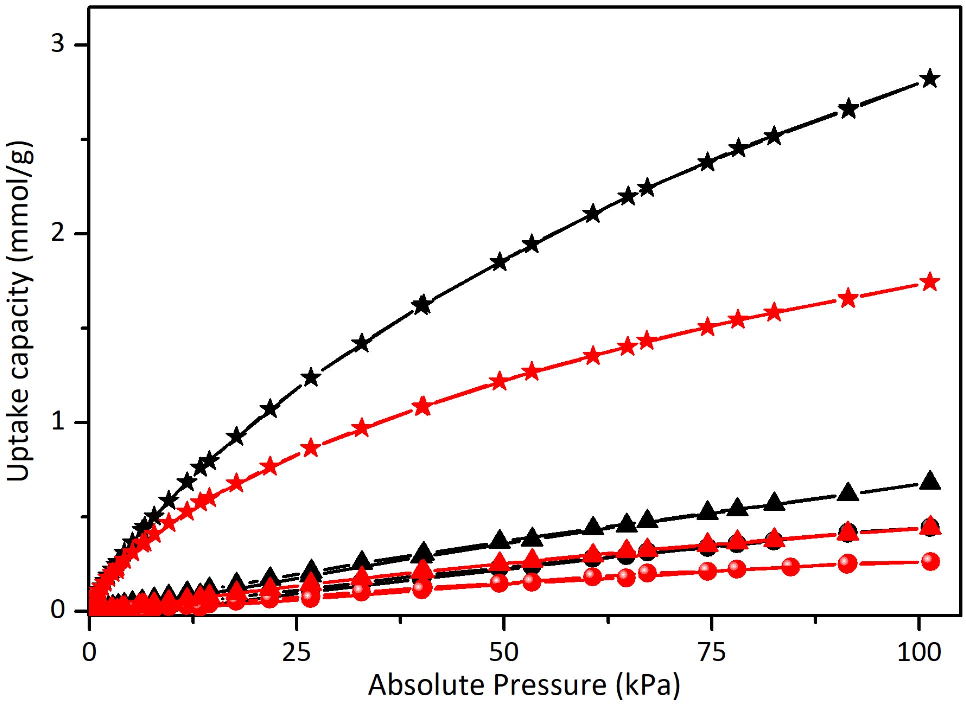

Figure S27. CH_4 uptake capacity isotherms of PPDA-BTCA-AGCOF (black line) and PPDA-BTCA-MCOF (red line). Uptakes were measured at 200 K (star-symbol), 273 K (triangle-symbol) and 298 K (sphere-symbol) at different pressures up to 100 kPa.

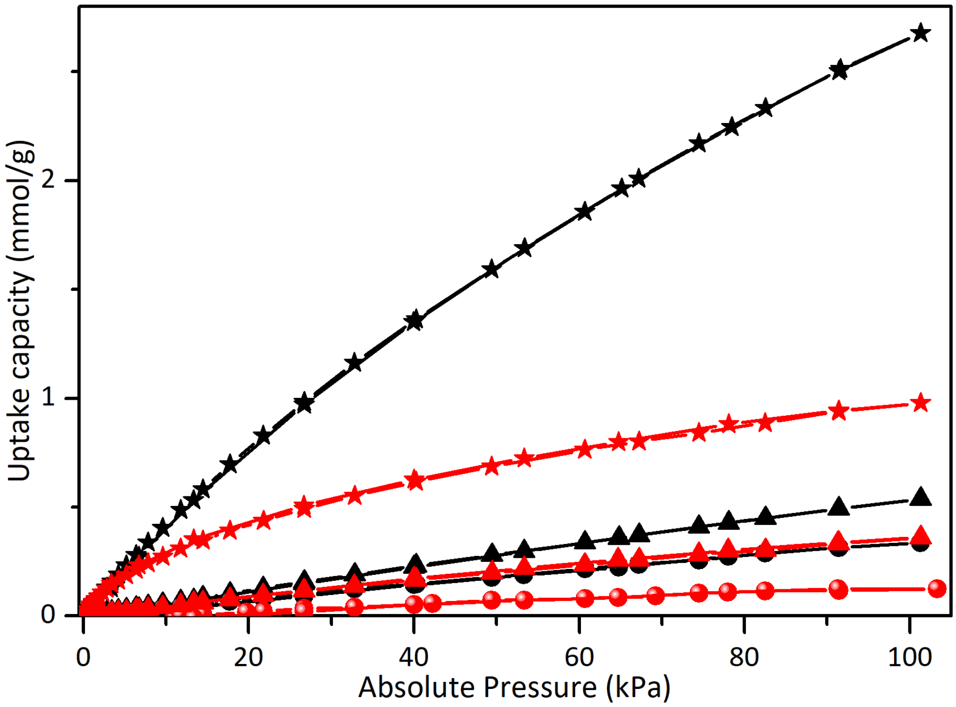

Figure S28. CH_4 **uptake capacity isotherms of TAPB-PDA-AGCOF (black line) and TAPB-PDA-MCOF (red line). Uptakes were measured at 200 K (star-symbol), 273 K (triangle-symbol) and 298 K (sphere-symbol) at different pressures up to 100 kPa.**

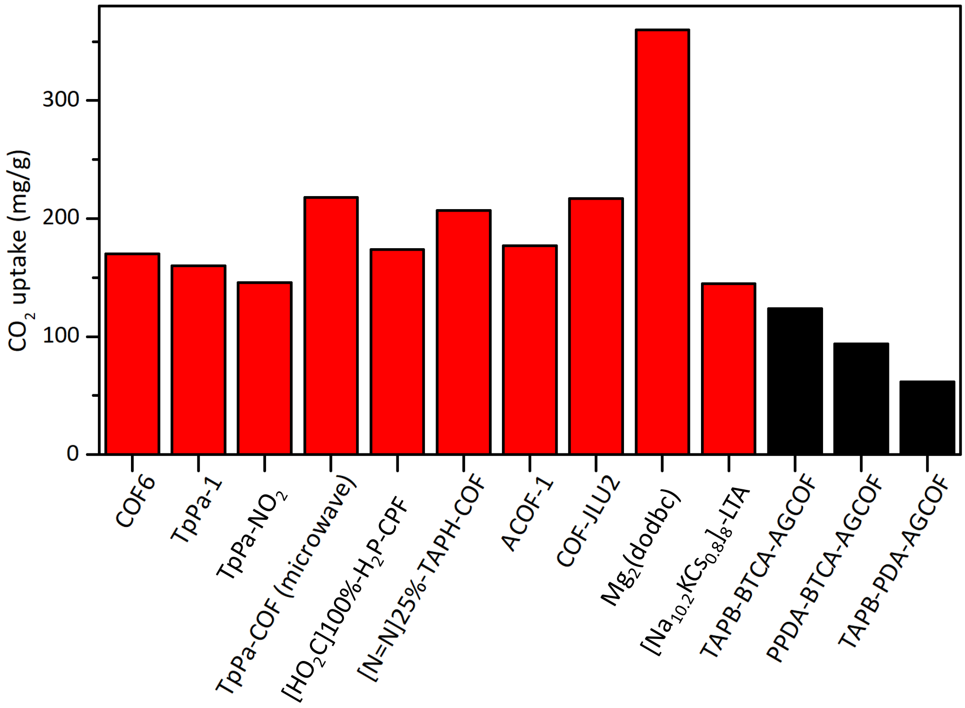

Figure S29. Summary of CO_2 **adsorption capacities reported for different COFs at 273 K, the best performance MOF (**Mg_2**(dodbc) at 298 K and zeolite ([**$Na_{10.2}KCs_{0.8}$**]-LTA at 1 bar. Herein reported samples are represented in black-columns.**

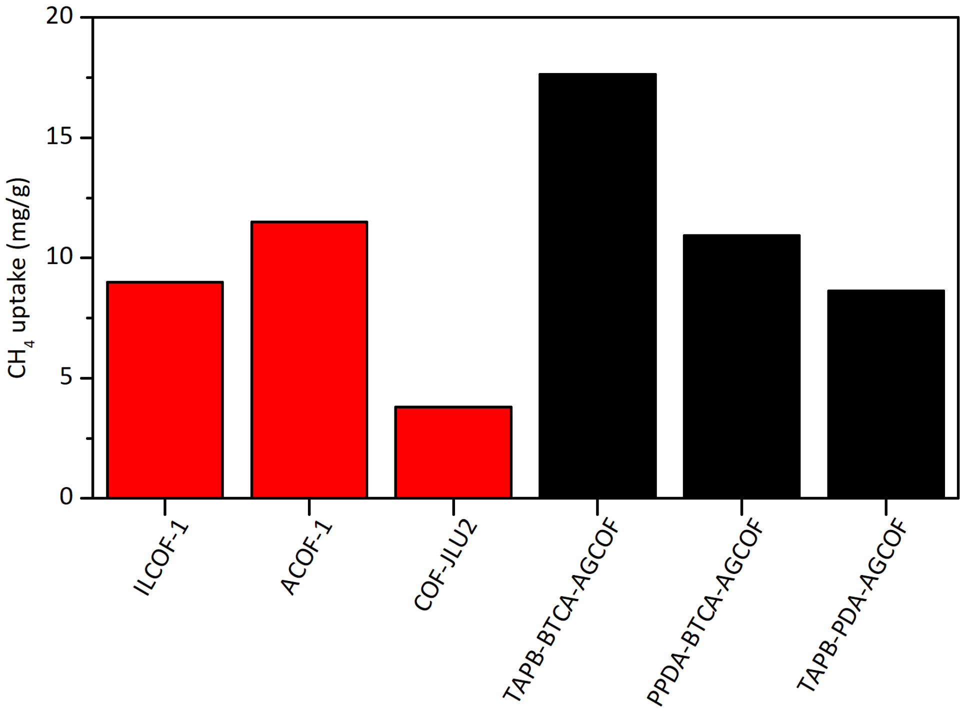

Figure S30. Summary of CH_4 adsorption capacities for the best 2D-COF at low pressure (1 bar). Herein reported samples are represented in black-columns.

Table S7. Summary of the uptake values for the COF-aerogels (AG) and COF-membranes (M).

	CO_2 298K			CO_2 273K			CO_2 200K		
	$cm^3\ g^{-1}$	$mmol\ g^{-1}$	$mg\ g^{-1}$	$cm^3\ g^{-1}$	$mmol\ g^{-1}$	$mg\ g^{-1}$	$cm^3\ g^{-1}$	$mmol\ g^{-1}$	$mg\ g^{-1}$
TAPB-BTCA-AG	54.0	2.41	106.0	63.0	2.81	123.6	305.2	13.6	599.1
TAPB-BTCA-M	27.3	1.22	53.5	49.0	2.19	96.14	181.6	8.10	355.6
PPDA-BTCA-AG	26.2	1.17	51.4	47.8	2.13	93.7	264.3	11.8	519.1
PPDA-BTCA-M	22.0	0.98	43.0	35.5	1.58	69.3	148.7	6.63	291.0
TAPB-PDA-AG	17.6	0.79	34.6	31.5	1.40	61.8	506.2	22.6	994.2
TAPB-PDA-M	13.7	0.61	26.8	23.9	1.07	47.0	120.9	5.40	237.5
	CH_4 298K			CH_4 273K			CH_4 200K		
	$cm^3\ g^{-1}$	$mmol\ g^{-1}$	$mg\ g^{-1}$	$cm^3\ g^{-1}$	$mmol\ g^{-1}$	$mg\ g^{-1}$	$cm^3\ g^{-1}$	$mmol\ g^{-1}$	$mg\ g^{-1}$
TAPB-BTCA-AG	17.97	0.80	12.86	24.63	1.09	17.63	84.26	3.76	60.31
TAPB-BTCA-M	6.43	0.28	4.49	13.91	0.62	9.94	41.26	1.84	29.51
PPDA-BTCA-AG	9.93	0.44	7.11	15.26	0.68	10.92	63.16	2.81	45.22
PPDA-BTCA-M	5.84	0.26	4.18	9.91	0.44	7.09	39.04	1.74	27.95
TAPB-PDA-AG	7.5	0.33	5.37	12.04	0.53	8.62	60.01	2.67	42.97
TAPB-PDA-M	2.75	0.12	1.96	8.06	0.36	5.77	21.92	0.97	15.69

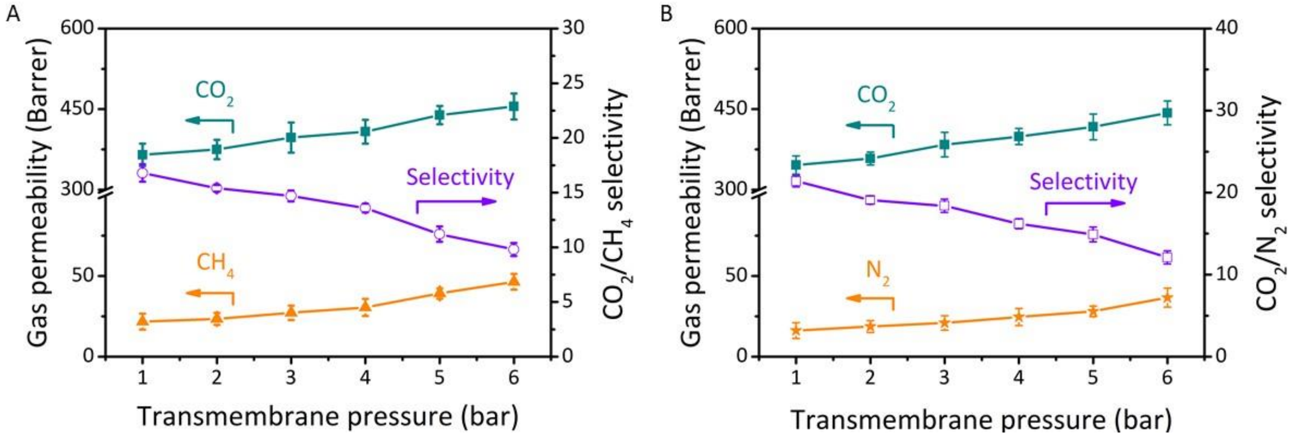

Figure S31. Study of the gas permeability and separation selectivity at different transmembrane pressures for (A) CO_2/CH_4 and (B) CO_2/N_2 in TAPB-BTCA-MCOF.

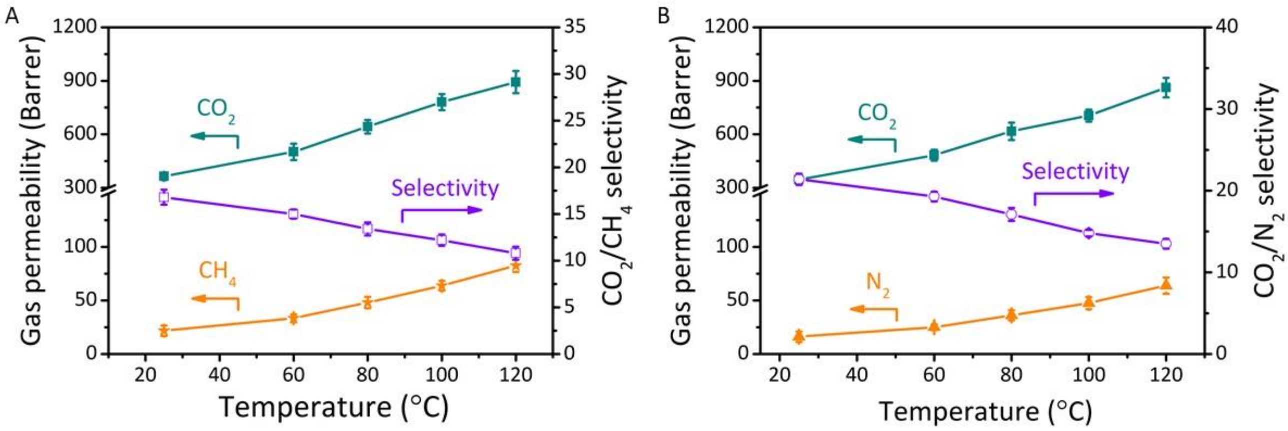

Figure S32. Study of the gas permeability and separation selectivity under different temperatures for (A) CO_2/CH_4 and (B) CO_2/N_2 in TAPB-BTCA-MCOF

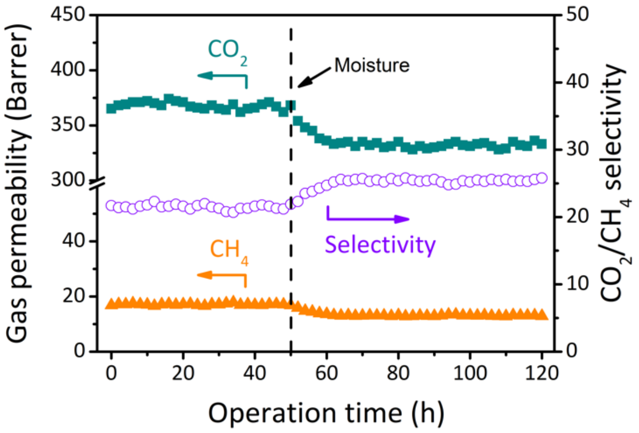

Figure S33. Evaluation of the long-term stability of TAPB-BTCA-MCOF for the separation of CO_2/CH_4 mixtures.

Table S8. CO_2/N_2 upper bound of commercial membranes and membranes made of MOFs, zeolites, porous organic polymers and COFs.

Group of membrane	Name	CO_2/N_2	CO_2 permeability (Barrer)	Reference
Commercial				
	PSf	23.2	5	[1]
	CA	26	6	[1b]
	PC	33	8.8	[1b]
	Matrimid	25.6	6.5	[1b]
MOF				
	ZIF-8	3	1900	[2]
	Bio-MOF-1	2.6	53000	[3]
	MOF-5	70	16700	[4]
Zeolite				
	MFI	18	30	[5]
	SAPO-34	5.6	1065	[6]
Porous organic				
	CC3	18.4	2520	[7]
COF				
	TAPB-BTCA-MCOF	21.4	346	This work
	PPDA-BTCA-MCOF	15.1	553	This work
	TAPB-PDA-MCOF	0.95	2460	This work

Table S9. CO_2/CH_4 upper bound of commercial membranes and membranes made of MOFs, zeolites, porous organic polymers and COFs.

Group of membrane	Name	CO_2/CH_4	CO_2 permeability (Barrer)	Reference
Commercial				
	PSf	26.1	5	[1]
	CA	29	6	[1b]
	PC	23.6	8.8	[1b]
	Matrimid	34	6.5	[1b]
MOF				
	ZIF-8	3.3	2640	[2]
	Bio-MOF-1	2.6	53000	[3]
	ZIF-90	1.5	1900	[8]
Zeolite				
	MFI	8	30	[5]
	SAPO-34	18.5	1065	[6]
	Ba-SAPO-34	103	4500	[9]
Porous organic				
	CC3	18.9	2520	[7]
COF				
	TAPB-BTCA-MCOF	16.8	365	This work
	PPDA-BTCA-MCOF	11.9	531	This work
	TAPB-PDA-MCOF	1.4	2466	This work

Table S10. Summary of the performance of COF-membranes for CO_2/CH_4 and CO_2/N_2 separation.

Name	Type	Substrate/matrix	Synthesis method	CO_2 permeability	SF CO_2/CH_4	SF CO_2/N_2	Ref.
$TpTG_B$-GO	Composite	PAN	LBL stacking	164.20 GPU	26.9		[10]
COF-LZU1-Pebax 1657	Composite	PAN	Transfer and dip coating	1752 GPU		25.1	[11]
TpPa-1	MMMs	PBI-BuI	Blending	13.1 (Barrer)	40.3	25.0	[12]
TpBD	MMMs	PBI-BuI	Blending	14.8 (Barrer)	48.7	23.0	[12]
ACOF-1	MMMs	Polyactive	Blending	98.9 (Barrer)		44	[13]
UiO-66-NH_2@TpPa-1	MMMs	PSf	Blending	7.1 (Barrer)	46.7		[14]
COF-5	MMMs	Pebax	Blending	493 (Barrer)		49.3	[15]
CTPP	MMMs	Pebax	Blending	73 (Barrer)	48.3	84.2	[16]
TpTAPM	MMMs	6FDA-DAM	Blending	850 (Barrer)	29.0		[17]
TpDAP	MMMs	6FDA-DAM	Blending	640 (Barrer)	27.6		[17]
PEG200@Dha-Tab	MMMs	Pebax	Blending	944 (Barrer)	33		[18]
ACOF-1	Pure	α-Al2O3	Solvothermal	56.64 GPU	97.1		[19]
TAPB-BTCA-MCOF	Pure		Pressure	365 (Barrer)	16.8	21.4	This work
PPDA-BTCA-MCOF	Pure		Pressure	531 (Barrer)	11.9	15.1	This work
TAPB-PDA-MCOF	Pure		Pressure	2460 (Barrer)	1.4	0.95	This work

SF. (Separation Factor), 1 GPU = 3.348×10^{-10} mol m^{-2} s^{-1} Pa^{-1}, 1 Barrer = 3.348×10^{-16} mol m^{-1} s^{-1} Pa^{-1}.

References

[1] a) M. S. Suleman, K. K. Lau, Y. F. Yeong, *J. Appl. Polym. Sci.* **2018**, *135* (1); b) Y. Zhang, J. Sunarso, S. Liu, R. Wang, *Int. J. Greenhouse Gas Control* **2013**, *12*, 84.

[2] N. A. H. Md Nordin, S. M. Racha, T. Matsuura, N. Misdan, N. A. Abdullah Sani, A. F. Ismail, A. Mustafa, *RSC Adv.* **2015**, *5* (54), 43110.

[3] J. A. Bohrman, M. A. Carreon, *Chem. Commun.* **2012**, *48* (42), 5130.

[4] Z. Zhao, X. Ma, A. Kasik, Z. Li, Y. S. Lin, *Ind. Eng. Chem. Res.* **2012**, *52* (3), 1102.

[5] a) C. Feng, K. C. Khulbe, T. Matsuura, R. Farnood, A. F. Ismail, *J. Membr. Sci. Res.* **2015**, *1* (2), 49; b) J. C. Poshusta, R. D. Noble, J. L. Falconer, *J. Membr. Sci.* **1999**, *160* (1), 115.

[6] S. Li, *J. Membr. Sci.* **2004**, *241* (1), 121.

[7] Q. Song, S. Jiang, T. Hasell, M. Liu, S. Sun, A. K. Cheetham, E. Sivaniah, A. I. Cooper, *Adv. Mater.* **2016**, *28* (13), 2629.

[8] A. Huang, Q. Liu, N. Wang, J. Caro, *Microporous Mesoporous Mater.* **2014**, *192*, 18.

[9] T. L. Chew, A. L. Ahmad, S. Bhatia, *Chem. Eng. J.* **2011**, *171* (3), 1053.

[10] M. Long, Y. Ma, C. Yang, R. Zhang, Z. Jiang, *J. Mater. Chem. A* **2021**, *9* (3), 1395.

[11] Y. Ying, Z. Yang, D. Shi, S. B. Peh, Y. Wang, X. Yu, H. Yang, K. Chai, D. Zhao, *J. Membr. Sci.* **2021**, *632*.

[12] B. P. Biswal, H. D. Chaudhari, R. Banerjee, U. K. Kharul, *Chemistry* **2016**, *22* (14), 4695.

[13] M. Shan, B. Seoane, E. Rozhko, A. Dikhtiarenko, G. Clet, F. Kapteijn, J. Gascon, *Chemistry* **2016**, *22* (41), 14467.

[14] Y. Cheng, Y. Ying, L. Zhai, G. Liu, J. Dong, Y. Wang, M. P. Christopher, S. Long, Y. Wang, D. Zhao, *J. Membr. Sci.* **2019**, *573*, 97.

[15] K. Duan, J. Wang, Y. Zhang, J. Liu, *J. Membr. Sci.* **2019**, *572*, 588.

[16] R. L. Thankamony, X. Li, S. K. Das, M. M. Ostwal, Z. Lai, *J. Membr. Sci.* **2019**, *591*.

[17] Y. Yang, K. Goh, P. Weerachanchai, T.-H. Bae, *J. Membr. Sci.* **2019**, *574*, 235.

[18] Y. Liu, H. Wu, S. Wu, S. Song, Z. Guo, Y. Ren, R. Zhao, L. Yang, Y. Wu, Z. Jiang, *J. Membr. Sci.* **2021**, *618*.

[19] H. Fan, A. Mundstock, J. Gu, H. Meng, J. Caro, *J. Mater. Chem. A* **2018**, *6* (35), 16849.
